# A digital intervention to support childhood cognition after the COVID-19 pandemic: a pilot trial

**DOI:** 10.1038/s41598-024-63473-2

**Published:** 2024-06-18

**Authors:** Hannah Kirk, Megan Spencer-Smith, Laura Jobson, Elizabeth Nicolaou, Kim Cornish, Ebony Melzak, Caitlin Hrysanidis, Cassie Moriarty, Belinda Davey, Theoni Whyman, Laura Bird, Mark A. Bellgrove

**Affiliations:** 1 School of Psychological Sciences and Turner Institute for Brain and Mental Health, Monash University, Melbourne, VIC Australia; 2 Faculty of Education, Monash University, Melbourne, VIC Australia; 3 Telethon Kids Institute, University of Western Australia, Perth, WA Australia

**Keywords:** Human behaviour, Therapeutics

## Abstract

Difficulties in executive functioning (EF) can result in impulsivity, forgetfulness, and inattention. Children living in remote/regional communities are particularly at risk of impairment in these cognitive skills due to reduced educational engagement and poorer access to interventions. This vulnerability has been exacerbated by the COVID-19 pandemic and strategies are needed to mitigate long-term negative impacts on EF. Here we propose a pilot trial investigating the benefits, feasibility, and acceptability of a school-based EF intervention for primary school students (6–8 years) living in regional, developmentally vulnerable, and socio-economically disadvantaged communities. Students were randomised to a digital intervention or teaching as usual, for 7 weeks. Children completed measures of EF and parents/educators completed ratings of everyday EF and social/emotional wellbeing at pre-intervention, post-intervention, and 3-month follow-up. Change in EFs (primary outcome), everyday EF, and social/emotional wellbeing (secondary outcomes) from pre- to post-intervention and pre-intervention to 3-month follow-up were examined. Feasibility and acceptability of the intervention was assessed through educator feedback and intervention adherence.

**Protocol Registration**: The stage 1 protocol for this Registered Report was accepted in principle on 20 April 2023. The protocol, as accepted by the journal, can be found at: 10.17605/OSF.IO/WT3S2. The approved Stage 1 protocol is available here: https://osf.io/kzfwn.

## Introduction

Switching attention, keeping track of activities, staying focused, and regulating emotions are critical skills required to support mental health, peer relationships, and academic success^1,2^. These skills are underpinned by multiple cognitive processes, collectively known as executive functions (EF). Core EFs consist of inhibition (i.e., the ability to control impulsive responding), working memory (i.e., the ability to temporarily store and mentally manipulate information) and cognitive flexibility (i.e., the ability to efficiently shift from one task to the next)^3,4^. A concerning proportion of primary-school students struggle to master EFs in the early school years placing them at risk of pervasive behavioural symptoms of inattention (e.g., distractibility, poor concentration), impulsivity, poor social and emotional development and learning difficulties^5,6^. Thus, disadvantaging them from the commencement of their education^5–7^.

In particular, children living in remote/regional communities are three times more likely to experience developmental problems across cognitive, behavioural, emotional and physical domains than their peers in metropolitan communities^8^. One fundamental factor that contributes to the disparities in the developmental trajectory of children across geographical locations is the lack of access to appropriate support services and educational resources^9,10^. Often there is a shortage of educators and specialists which can result in long wait times and extensive travel to access quality resources^8,11^. This is concerning given that 3.4 billion people globally reside in rural areas^12^.

Further, the suspension of face-to-face classes in schools during the COVID-19 pandemic has led to concerns regarding student’s development and learning^13,14^. Despite massive efforts by educators and schools, evidence suggests significant underperformance in cognitive and academic domains in primary school children as a result of school closures, and worryingly highlights that diminished performance is larger for students who are already disadvantaged^15^. This includes students from families with low socio-economic status, and those living in remote/regional communities where it is likely that the extra challenges of remote learning have compounded existing inequities^16^. Worryingly individuals who are already at risk of poor outcomes due to pre-existing EF deficits appear to be more vulnerable to the negative impact of the COVID-19 pandemic on several key health behaviours such as physical activity^17^. Of additional concern is early data that indicates longer school closures are associated with more significant negative impacts on learning^13^. Students living in the state of Victoria, Australia have spent more time out of the classroom (approx. 200 days of lost in-class learning) since the pandemic started than most other students in the world^18^. As such, they may be at particular risk of the negative impacts of the pandemic. According to the Australian Early Development Census (AEDC^19^) which assesses childhood developmental vulnerability across physical, social, emotional, cognitive and communication domains at school entry, 1 in 5 Australian children were identified as being developmentally vulnerable (i.e., lowest 10 per cent in at least one of the five domains) prior to the COVID-19 pandemic. The risk of developmental vulnerability increased the further children lived from cities, and children living in the most disadvantaged socio-economic areas were three times more likely to experience vulnerability in more than one domain than children in the most advantaged socio-economic areas^19^. By their third year of schooling children identified as at risk of developmental vulnerability were typically a year behind their peers on standardised academic testing measures (e.g., National Assessment Program—Literacy and Numeracy (NAPLAN)). It is therefore imperative that tailored and sustained support is provided to ensure students can readjust and catch-up on lost learning and cognitive development.

Overwhelming evidence highlights that resources that are delivered early, when core foundational skills such as EFs are still undergoing critical growth, are the most effective at providing sustained impacts on a child's developmental trajectory^20^. Given that EFs support short- and long-term academic outcomes as well as social/emotional well-being^7,21^, resources that aim to strengthen these skills in early education may assist in minimising the widening gap between students at risk of developmental vulnerability due to geographical and environmental factors, and their urban peers^22,23^. Interventions that have attempted to promote EF in childhood range from behaviour-focused interventions whereby teachers set rules and discipline strategies^24^, social-emotional learning interventions which teach emotional knowledge and social problem-solving skills^24^, and academic skills (i.e., maths and reading) interventions^25^. A recent review of these existing intervention approaches indicated positive impacts on areas such as classroom management, behavioural regulation and social behaviours in preschool children but smaller effects for improvements in EF^26^. One intervention approach that aims to target core cognitive skills, including EF directly, is digital cognitive training. This approach, which involves repeated practice on certain cognitive tasks, has seen a surge in popularity over the past 20 years. Unlike existing resources that are largely designed for the clinical childhood population (i.e., psychopharmacology and neurofeedback) and thus outside the scope of educational settings, digital training interventions are easy to administer to groups of children, affordable and scalable, such that they have the potential to overcome existing geographical and sociocultural barriers. Indeed, digital approaches are rapidly becoming one of the most prominent modalities to deliver additional support to students in the classroom. Studies that have evaluated digital school-based training of EFs have consistently demonstrated training gains (i.e., improvement in performance in the training tasks from the first to last training session) and near-transfer (i.e., change on tasks structurally similar to the training tasks pre-to-post training; Refs.^27–29^). In a meta-analysis of 32 studies, cognitive training interventions were shown to improve EFs in preschool children, with children from families experiencing low socio-economic status experiencing the greatest benefits from training^30^. However, there is inconsistent evidence that this type of training can generalise to improvements in classroom behaviour or outcomes that differ from the trained processes (i.e., far transfer; Ref.^30^).

Although inconsistent transfer effects observed across training studies are largely attributed to methodological concerns, such as inadequate sample size and inappropriate assessment measures, recent reviews suggest that the design of the training interventions themselves may also play a role^31^. Most existing training programs focused almost exclusively on training one cognitive domain (most commonly working memory^32^). However, reviews indicated that this approach may be significantly enhanced by multi-domain training where several cognitive processes are targeted^33–37^. Emerging evidence demonstrates that multi-domain training also has impressive advantages over single-domain training in maintaining intervention effects^38,39^. Furthermore, recent research indicated that game-based training tasks might be more effective than standard training tasks^40^. Gamified cognitive training may promote greater effort and engagement during training, which may result in greater gains post training compared to non-gamified cognitive training interventions^41,42^. Therefore, gamified training that targets multiple cognitive domains and can be implemented in early educational settings could offer the potential to promote both near and far skill transfer in children living in areas with high rates of developmental vulnerability.

In addition to the mode of intervention delivery and the targeted cognitive domain, the developmental stage in which these interventions are delivered is also likely to play a crucial role in their effectiveness. The majority of EF interventions have focused on preschool students due to rapid growth in EF processes from ages 3 to 5 years^43^. However, recent reviews suggest EF interventions may be more effective when children have more established cognitive skills that allow them to employ cognitive strategies more deliberately^26^. Although no empirical study to date has directly investigated the timing of intervention delivery in relation to developmental stage, investigations of predictors of cognitive training outcomes more broadly suggest that a certain level of general cognitive resources are required to effectively engage with the intervention^44,45^. Collectively these studies suggest that EF interventions may be beneficial in primary school students.

Primary education settings offer an optimal context to deliver interventions as they provide continuity of care and ensure equitable intervention access and support for all students^46^. These factors are particularly pertinent for students living in remote/regional communities who often have limited access to appropriate resources in the community. In addition, the proliferation of digital interventions offers the potential to increase accessibility and viability of interventions in remote/regional settings by reducing the need for in-person support which can be costly, impractical and unsustainable. Despite potential benefits, previous studies have identified challenges in delivering digital interventions remotely in the classroom, with concerns from educators centring around the time and resources required to upskill to implement specific technology^47^. Given the lack of research in this area it is crucial to evaluate the delivery of EF interventions remotely in educational settings in order to determine feasibility and contextualise how geographical factors may influence implementation success. This trial aimed to pilot a game-based multi-domain digital training intervention (Caterpillar Creek) co-designed with developmental and clinical psychologists, neuroscientists, cross-cultural researchers, primary school students and educators across the state of Victoria, Australia and aimed at strengthening EF in students living in regional areas with high rates of childhood developmental vulnerability and socio-economic disadvantage. The intervention has been developed in line with the ORBIT model^48^ which involves an iterative process with pre-specified milestones for progression from design (Phase I) to preliminary testing (Phase II), efficacy trials (Phase III) and effectiveness trials (Phase IV). In this pilot randomised controlled trial our primary aim was to conduct preliminary testing (Phase II) to assess whether the digital multi-domain training intervention promotes change in key EFs (inhibitory control, working memory and cognitive flexibility) from pre-to post-intervention (i.e., immediate near transfer) compared to a teaching as usual control condition, in primary school students living in regional areas with high rates of childhood developmental vulnerability and socio-economic disadvantage. Our secondary aims included examining whether the intervention promotes change in functional skills such as everyday EFs and social/emotional wellbeing (i.e., immediate far transfer); and promotes sustained change in EFs or functional skills from pre-intervention to 3-month post-intervention follow-up (i.e., sustained near/far transfer). In addition, we aimed to assess the feasibility and acceptability of the program implemented via the school classroom. The specific research questions and hypothesis of this pilot trial are outlined in Table 1.

**Table 1:** Design Table. Each research question, hypothesis, and sampling/analysis plans for the pilot trial.

Question	Hypothesis	Sampling plan (e.g., power analysis)	Analysis plan	Interpretation given to different outcomes
Primary outcome				
Does the digital multi-domain training intervention promote change in inhibitory control (response inhibition) from pre-intervention to post-intervention compared to UT?	H1: Students who receive the intervention will experience greater change in response inhibition performance (as measured by SSRT on the OSARI task) from pre (T1) to post-intervention (T2) compared to students who continue with UT H0: Change in response inhibition performance from T1 to T2 is unrelated to group (intervention vs UT)	Previous trials indicate the lowest estimate of effect size to be medium between our EF intervention and UT. Power calculations suggest 210 students would provide 95% power to detect a medium effect size in our outcome measures. For practical reasons, sample size is confined to 140 which will provide 84% power to detect a medium effect. We will stop sampling participants when either 140 participants have been recruited OR at the latest date possible for participants to complete T1 and T2 assessments within the current school year	A latent change score model will be estimated to assess any change in the primary outcome measure of response inhibition (SSRT on the OSARI) between T1 to T2 (short term effects) as a function of group (intervention vs UT). Age will be controlled for as a confounder in the model	The results are consistent with H1 if students who received the intervention show significantly greater change in response inhibition from T1 to T2 compared to students who continue with UT (*BF*_10_ ≥ 3) The results are consistent with the null hypothesis if no interaction between group and change from T1 to T2 is found (*BF*_01_ ≥ 3 A Bayes factor (*BF*) will be calculated to quantify the strength of evidence for the null versus the H_1_ model
Secondary outcomes				
Does the digital multi-domain training intervention promote change in inhibitory control (interference control) from pre-intervention to post-intervention compared to UT?	H2: Students who receive the intervention will experience greater change in interference control performance (as measured by the Conflict Score on the Child ANT) from T1 to T2 compared to students who continue with UT H0: Change in interference control performance from T1 to T2 is unrelated to group (intervention vs UT)	Previous trials indicate the lowest estimate of effect size to be medium between our EF intervention and UT. Power calculations suggest 210 students would provide 95% power to detect a medium effect size in our outcome measures. For practical reasons, sample size is confined to 140 which will provide 84% power to detect a medium effect. We will stop sampling participants when either 140 participants have been recruited OR at the latest date possible for participants to complete T1 and T2 assessments within the current school year	A latent change score model will be estimated to assess any change in the outcome measure of interference control (conflict score on the Child ANT) between T1 to T2 (short term effects) as a function of group (intervention vs UT). Age will be controlled for as a confounder in the model	The results are consistent with H2 if students who received the intervention show significantly greater change in interference control from T1 to T2 compared to students who continue with UT (*BF*_10_ ≥ 3 ) The results are consistent with the null hypothesis if no interaction between group and change from T1 to T2 is found (*BF*_01_ ≥ 3). A Bayes factor (*BF*) will be calculated to quantify the strength of evidence for the null versus the H_1_ model
Does the digital multi-domain training intervention promote change in working memory from pre-intervention to post-intervention compared to UT?	H3: Students who receive the intervention will experience greater change in working memory performance (as measured by the Total Score on Verbal and Visuospatial Serial Span Tasks) from T1 to T2 compared to students who continue with UT H0: Change in working memory performance from T1 to T2 is unrelated to group (intervention vs UT)	Previous trials indicate the lowest estimate of effect size to be medium between our EF intervention and UT. Power calculations suggest 210 students would provide 95% power to detect a medium effect size in our outcome measures. For practical reasons, sample size is confined to 140 which will provide 84% power to detect a medium effect. We will stop sampling participants when 140 participants have been recruited OR at the latest date possible for participants to complete T1 and T2 assessments within the current school year	A latent change score model will be estimated to assess any change in the outcome measures of working memory (verbal and visuospatial) between T1 to T2 (short term effects) as a function of group (intervention vs UT). Age will be controlled for as a confounder in the model	The results are consistent with H3 if students who received the intervention show significantly greater change in working memory from T1 to T2 compared to students who continue with UT (*BF*_10_ ≥ 3) The results are consistent with the null hypothesis if no interaction between group and change from T1 to T2 is found (*BF*_01_ ≥ 3). A Bayes factor (*BF*) will be calculated to quantify the strength of evidence for the null versus the H_1_ model
Does the digital multi-domain training intervention promote change in cognitive flexibility from pre-intervention to post-intervention in primary school students at risk of developmental vulnerability, compared to UT?	H4: Students who receive the intervention will experience greater change in cognitive flexibility performance (as measured by total errors on the WCST and total number of advanced ‘responses on the DCCS Task) from T1 to T2 compared to students who continue with UT H0: Change in cognitive flexibility performance from T1 to T2 is unrelated to group (intervention vs UT)	Previous trials indicate the lowest estimate of effect size to be medium between our EF intervention and UT. Power calculations suggest 210 students would provide 95% power to detect a medium effect size in our outcome measures. For practical reasons, sample size is confined to 140 which will provide 84% power to detect a medium effect. We will stop sampling participants when 140 participants have been recruited OR at the latest date possible for participants to complete T1 and T2 assessments within the current school year	A latent change score model will be estimated to assess any change in the outcome measures of cognitive flexibility (matching and switching) between T1 to T2 (short term effects) as a function of group (intervention vs UT). Age will be controlled for as a confounder in the model	The results are consistent with H4 if students who received the intervention show significantly greater change in cognitive flexibility from T1 to T2 compared to students who continue with UT (*BF*_10_ ≥ 3) The results are consistent with the null hypothesis if no interaction between group and change from T1 to T2 is found (*BF*_01_ ≥ 3). A Bayes factor (*BF*) will be calculated to quantify the strength of evidence for the null versus the H_1_ model
Does the digital multi-domain training intervention promote change in everyday EF and social/emotional wellbeing from pre-intervention to post-intervention compared to UT?	H5: Changes in everyday EF (as measured by the GEC score on the BRIEF-2) and social/emotional wellbeing (as measured by the Total Score on the SDQ) from T1 to T2 is unrelated to group (intervention vs UT)	Previous trials indicate the lowest estimate of effect size to be medium between our EF intervention and UT. Power calculations suggest 210 students would provide 95% power to detect a medium effect size in our outcome measures. For practical reasons, sample size is confined to 140 which will provide 84% power to detect a medium effect. We will stop sampling participants when 140 participants have been recruited OR at the latest date possible for participants to complete T1 and T2 assessments within the current school year	A latent change score model will be estimated to assess any change in the outcome measures of functional skills (everyday EF and social/emotional wellbeing) between T1 to T2 (short term effects) as a function of group (intervention vs UT). Age will be controlled for as a confounder in the model	The results are consistent with H5 if no interaction between group and change from T1 to T2 is found (*BF*_01_ ≥ 3). A Bayes factor (*BF*) will be calculated to quantify the strength of evidence for the null versus the H_1_ model
Does the digital multi-domain training intervention promote sustained change in any primary or secondary outcomes from pre-intervention to 3-month follow-up compared to UT?	H6a: Students who receive the intervention will experience greater change in response inhibition performance (primary outcome) from T1 to T3 compared to students who continue with UT H0: Changes in response inhibition from T1 to T3 is unrelated to group (intervention vs UT) H6b: Changes in secondary outcomes from T1 to T3 is unrelated to group (intervention vs UT)	Previous trials indicate the lowest estimate of effect size to be medium between our EF intervention and UT. Power calculations suggest 210 students would provide 95% power to detect a medium effect size in our outcome measures. For practical reasons, sample size is confined to 140 which will provide 84% power to detect a medium effect. We will stop sampling participants when 140 participants have been recruited OR at the latest date possible for participants to complete T1 and T2 assessments within the current school year	A latent change score model will be estimated to assess any change on any of the outcome measures between T1 to T3 (stability effects) as a function of group (intervention vs UT). Age will be controlled for as a confounder in the model	The results are consistent with H6a if students who received the intervention show significantly greater change in response inhibition from T1 to T3 compared to students who continue with UT (*BF*_10_ ≥ 3). The results are consistent with the null hypothesis if no interaction between group and change in response inhibition from T1 to T3 is found (*BF*_01_ ≥ 3) The results are consistent with H6b if no interaction between group and change on any of the secondary outcome measures from T1 to T3 is found (*BF*_01_ ≥ 3). A Bayes factor (*BF*) will be calculated to quantify the strength of evidence for the null versus the H_1_ model
Is the digital multi-domain training intervention feasible and acceptable to implement in the primary school classroom curriculum?	H7: Compliance with the intervention will be high (> 80%) and educators will rate the program as feasible and acceptable (mean score > 3 on the feasibility survey)	This research question does not assess efficacy of the intervention and therefore does not assess change over time nor change across groups. As such a sample of 140 students is sufficient to test the proposed hypothesis	Mean compliance rates will be calculated across all students assigned to the intervention. Mean scores on the educator feasibility survey will be calculated	The results are consistent with H7 if compliance with the intervention is on average ≥ 16 out of 20 sessions, and the educator feasibility score is greater on average than 3

*UT* usual teaching, *T1* pre-intervention, *T2* post-intervention, *T3* 3-month follow-up, *SSRT* stop signal reaction time, *OSARI* open-source anticipatory response inhibition task, *ANT* attention network task, *WCST* Wisconsin card sorting task, *DCCS* dimensional change card sort, *EF* executive functioning, *GEC* global executive composite, *BRIEF* behavior rating inventory of executive function, *SDQ* strengths and difficulties questionnaire.

## Methods

### Ethics information

The study was approved by the Monash University Human Research Ethics Committee on 19 December 2019 (#22,515). The study was also approved by the Department of Education and Training on 4 February 2020 (#2019_004263). The pilot trial will be conducted and reported in accordance with the *Consolidated Standards of Reporting Trials (CONSORT) Statement: Extension to Randomised Pilot and Feasibility Trials*^49^ and has been prospectively registered with the Australian New Zealand Clinical Trial Registry (ACTRN: 12622000877785; 21st June 2022). Informed consent will be obtained from all participant caregivers and educators.

### Design and study setting

This study is a pilot randomised controlled trial with two parallel groups. The study was conducted in Australian government and independent primary schools across Victoria from July 2022 to December 2022. The effects of the school-based intervention compared with a teaching as usual control condition was assessed at pre-intervention (week 1), immediately post-intervention (week 9), and at 3-months post-intervention (week 22).

### Eligibility criteria

Primary schools located in remote/regional communities in the state of Victoria, Australia with (a) high levels of childhood developmental vulnerability as defined by the Australian Early Development Census (AEDC 2018; ≥ 80th percentile) and/or (b) socio-economic disadvantage based on the national Socio-Economic Indexes for Areas (SEIFA, Index of Relative Socio-economic Advantage and Disadvantage ≤ 950) were considered eligible. Within these schools, students were eligible to participate if they were in Grade 1 or 2 classes.

### Randomisation and blinding

Participating students within Grade 1 and 2 classes in participating schools completed either a digital EF training intervention or continued with their usual classroom teaching. Although the inclusion of a placebo control condition is desirable, the school-based setting of the trial means that implementing a placebo program during class time (which would replace existing educational content) is unethical. Randomisation occurred at the individual level and was computer generated using a 1:1 ratio within blocks of 10. Allocation concealment was maintained by keeping randomisation records in a secure location that is only accessible to one member of the research team. This unblinded member of the research team performed a balance check once 50% of the target sample had been enrolled in the trial to ensure balance on key demographic characteristics (i.e., age, gender) across groups. If the groups were unbalanced then re-randomisation occurred until balance was achieved. Data collectors and data analysts were blinded to group allocation for the entirety of the trial including data analysis. Any unblinding events were recorded, noting the time and reason for the unblinding.

### Procedure

Researchers blinded to the allocation of students administered computerised assessment measures of EF at pre-intervention, post-intervention, and at 3-month follow up. These assessments were conducted in-person in groups (< 3 students) in a quiet room at the student’s school (~ 1–1.5 h). Parents/caregivers and educators completed online questionnaires via an email link at each of the assessment timepoints to estimate the student’s everyday executive functioning, and social/emotional wellbeing (~ 20–30 min). At pre-intervention students completed a verbal and nonverbal intelligence test and parent/caregivers completed a demographic survey (see Table 2 for a schedule of measures). Students randomised to the intervention were instructed to use the program 2–3 times a week during class time for seven consecutive weeks (20 training sessions, each ~ 30 min in duration). Educators were provided with an instruction manual and an initial training session to ensure they were confident in administering and supervising students over the course of the intervention. Educators were contacted by a member of the research team once a week to track progress and troubleshoot any queries. Each student was provided with a Samsung Galaxy tablet (10.1 inches), a unique username to access the program, and individual headphones. After the post-intervention assessment students assigned to the intervention returned the study tablets and headphones and recommenced their usual teaching. At post-intervention educators responded to an online survey regarding intervention feasibility, and parents responded to an online survey regarding perceived intervention benefits.

### Digital cognitive training intervention

**Figure 1:**
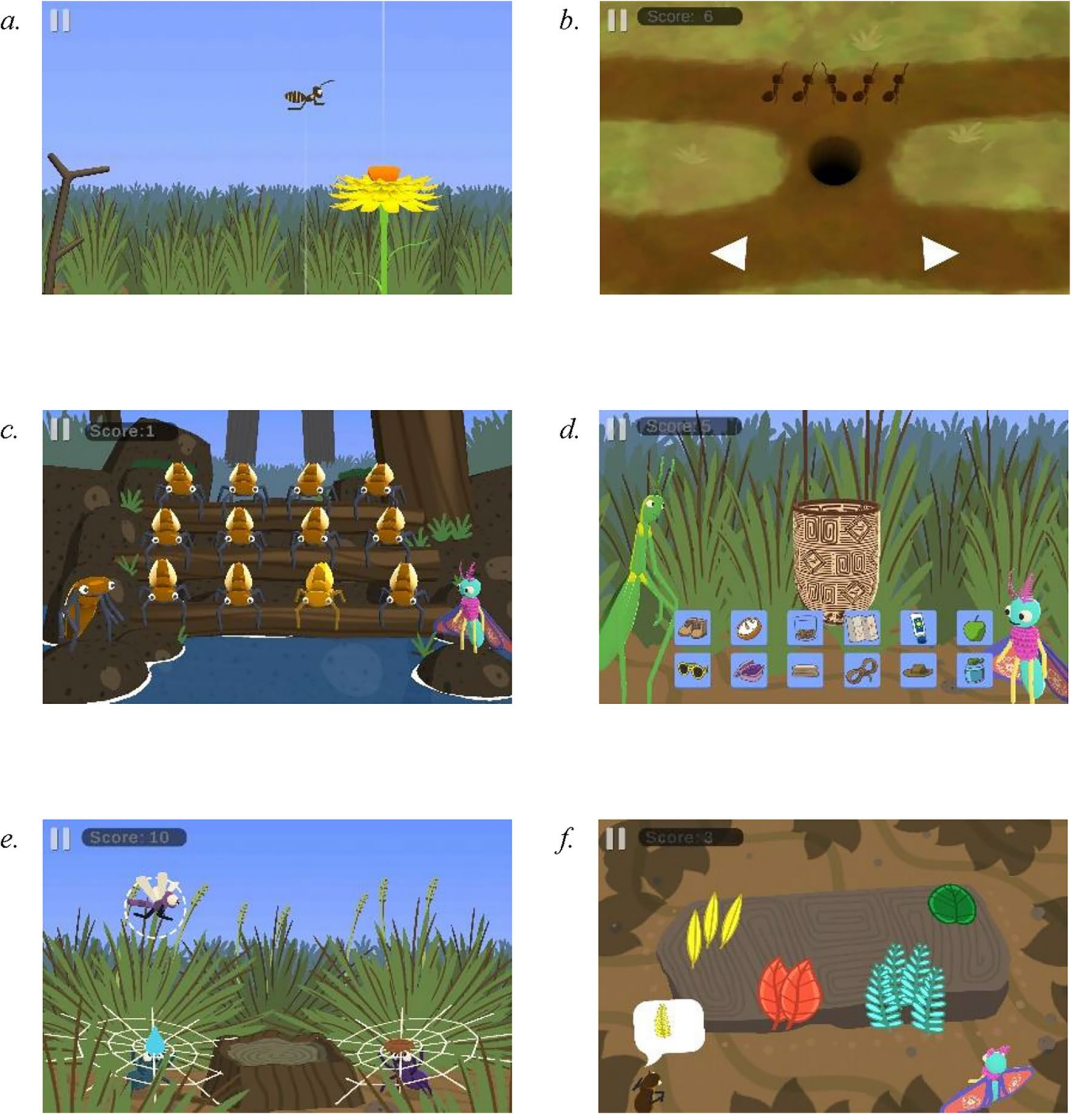
Caterpillar Creek Training Exercises. The two inhibitory control exercises target (**a**) response inhibition and (**b**) interference control. The two working memory exercises target (**c**) visuospatial working memory and (**d**) verbal working memory. The two cognitive flexibility exercises target (**e**) switching and (**f**) matching.

Caterpillar Creek is a digital training intervention designed specifically for this study with the intention of improving inhibitory control, working memory, and cognitive flexibility in primary school students aged 6 to 8 via 6 adaptive game-based exercises (see Fig. 1). The intervention was co-designed through a series of focus groups with educators and students across regional and metropolitan areas in the state of Victoria, Australia (see Supplementary Information; Phase 1) and was developed to be delivered in classrooms over the course of a school term. To ensure elements of Aboriginal culture were appropriately incorporated into the intervention an Aboriginal researcher, community elder, graphic designer and musician were involved in the design of the intervention (see Supplementary Information; Phase 1). The gamified intervention is set within a sparse landscape (Caterpillar Creek) that has been damaged by a storm. Students are instructed to restore the land and help feed its inhabitants (e.g., caterpillars) by completing exercises and obtaining rewards (e.g., seeds, water, and leaves). Students complete a total of 20 training sessions, with each training session comprising 6 exercises, each 3 min in duration (total active training time of 18 min). The order in which students complete the exercises are randomised each day.

Inhibitory control was trained through two exercises: (1) a *Response Inhibition* exercise where students are presented with a target point (a flower) on the far right of the screen. Students are required to press and hold a button on the screen, resulting in a bee flying from the left to the right of the screen. They must lift their finger off the button as close to the target as possible, allowing the bee to land on the flower. Sometimes the target location will become unavailable before the bee reaches it (i.e., flower closes; stop-signal; 25% of trials), and the student is required to keep holding the button allowing the bee to safely fly off the screen. This exercise adapts in difficulty in real-time based on the students’ inhibitory performance by means of a staircase algorithm method^50^. If students fail to inhibit responding to a stop-signal (i.e., closed flower) then the time between the button being pressed and the stop-signal occurring (stop signal delay: SSD) in the subsequent trial will be reduced by 50 ms. If students correctly inhibit responding to a stop-signal, then the SSD will increase by 50 ms for the subsequent trial. This exercise is based on stop-signal tasks which are the gold-standard neuropsychological tests of response inhibition^51–53^. (2) In an *Interference Control* exercise, students must make a directional response (left or right) depending on the orientation of a central target. They are required to ignore distractors that appear on either side of the target. These distractors face in either the same (congruent) or opposite (incongruent) direction as the target. The exercise adapts in difficulty by increasing the number of distractors and by delaying the onset of the target after the distractors have appeared. This exercise is based on classic flanker tasks used to assess interference control^54,55^.

Working memory was trained through the following exercises: (3) a *Visuo-Spatial Serial Recall* exercise which requires students to remember and then recall the order and spatial location of targets that light up on the screen. (4) A *Verbal Serial Recall* exercise which requires the student to remember a verbally presented list of items and then recall this list in backwards ordering by selecting images of the items from a bank of items on the screen. Both exercises adapt by increasing the number of items to be remembered. These exercises were designed based on established neuropsychological assessments of working memory (e.g., spatial pointing and digit span tasks; Refs.^44,56^).

Cognitive flexibility was trained through the following exercises: (5) a *Switching* exercise in which students are presented with four targets (i.e., purple; yellow; purple striped and yellow striped dragonflies) and are required to respond to each target by pressing a left or right button depending on a predefined rule. The sorting rules are either one-dimension (i.e., purple dragonflies = left; yellow dragonflies = right) or two-dimension (i.e., yellow striped + purple plain dragonflies = left; yellow plain + purple striped dragonflies = right). In more advanced levels, events occur (i.e., windstorms) that temporarily reverse the sorting rules. This exercise was based on gold-standard cognitive flexibility tasks such as the Dimensional Change Card Sort test^57^. (6) The final exercise is a *Matching* exercise in which students are presented with a target in addition to four other items. Students are required to match the target to one of the four items based on colour, quantity, or shape. Students are not informed of the correct sorting dimension prior to the trial so need to work this out through trial and error. This exercise adapts by increasing the frequency that the sorting dimension changes (i.e., after 12, 8, 6, 4 consecutive correct responses). This exercise was based on switching tasks such as the Wisconsin Card Sorting Test^58^.

### Demographic measures

*General Cognitive Functioning* of students was assessed using the Kaufman Brief Intelligence Test—2^nd^ Edition [KBIT 2; Ref.^59^]. The KBIT-2 takes 15 min to administer and is suitable for individuals aged 4 to 90 years. The battery has three subscales: Verbal Knowledge, Matrices and Riddles and produces a verbal, non-verbal and composite IQ score. The full-scale IQ composite score was used to confirm eligibility in the trial (> 70) and was used as a covariate in analyses. Test–retest reliability ranges from 0.88 to 0.92^60^.

*Family Socio-Demographic Information* including caregivers’ highest education level, marital status, employment status, occupation, and household circumstances was collected at pre-intervention via an online demographic survey completed by parent/caregivers of participating students.

*Intrinsic Motivation* of students was assessed using the Cognitive Persistence subscale of the Revised Dimensions of Mastery Questionnaire (DMQ; Ref.^61^) parent-report for school-age children. The Cognitive Persistence subscale consists of 6 items which parents’ rate on a 5-point Likert scale (1 = not at all like this child to 5 = exactly like this child). A total score was obtained by summing all item scores and dividing by the number of items in the scale. Higher scores indicate greater motivation.

*Family Functioning* was assessed using the General Functioning subscale of the McMaster Family Assessment Device (FAD^62^). Parents rated 12 items and decided how well each statement described their own family on a scale of 1 (strongly disagree) to 4 (strongly agree). The FAD was scored by totalling all item scores and dividing by the number of items in the scale. Higher scores indicate poorer levels of family functioning.

### Primary outcome measure|Near transfer

*Inhibitory Control (Response Inhibition)* was assessed using the Open-Source Anticipated Response Inhibition Task (OSARI; Ref.^52^). The parameters of the task were guided by the Stop Signal Consensus Guide^51^. Students faced a computer monitor and were presented with a 3 cm × 15 cm vertical rectangular bar in the centre of the screen. A ‘target’ point was located at 80% of the total bar height. Children were instructed to use the index finger on their dominant hand to press and hold the space key to initiate a trial. The bar then filled up from the bottom to the top at a constant speed. Go trials require students to lift their finger off the button when the bar was filled to the ‘target’ point (at 800 ms). On Stop trials, the bar spontaneously stopped filling up between 300 and 700 ms (i.e., Stop Signal Delay [SSD]) and students were required to keep their finger on the button until the bar is filled (at 1000 ms). The spontaneous stopping of the bar filling up (i.e., Stop cue) initially occurred at 500 ms into the trial and then decreased or increased by 50 ms depending on trial success or failure, respectively. Students completed 10 practice Go trials and 20 randomly presented practice Go and Stop trials (15 Go and 5 Stop trials). Once students completed the practice trials, they then completed three test blocks. Each test block contained a total of 60 randomly presented trials, with 45 (75%) being Go trials and 15 (25%) being Stop trials. A total of 180 trials were completed (135 Go trials and 45 Stop trials). The main variable of interest is the Stop Signal Reaction Time (SSRT; calculated using the integration method). Other variables of interest are Go trial accuracy (%), Go trial reaction time (RT), RT variability and maximum SSD. Performance cut-offs were applied to exclude data indicating lack of task understanding, e.g., < 75% Go trial response rate, < 60% accuracy on Go-trials, or RTs < 400 ms^51,52^. Anticipated response (AR) paradigms of the stop-signal task have been shown to offer more reliable SSRT estimates than choice response and simple response time versions of the stop-signal paradigm^63^. In addition, as AR paradigms restrict the possible range of RTs on Go-trials, the risk of strategic slowing, which is common in stop signal tasks is mitigated^63^.

### Secondary outcome measures|Near transfer

*Inhibitory Control (Interference Control)* was assessed using the Child Attention Network Task (ANT^55^). Students were seated in front of a laptop and an external keyboard. Students were presented with a fixation cross in the centre of the screen (400–1600 ms randomised), followed by a single horizontal fish or a row of five horizontal fish presented above or below a fixation point. Students were required to report the direction in which the central target fish is pointing by pressing either the right or left key on the keyboard with their index finger. The two fish on either side (flankers) point in either the same (congruent) or opposite (incongruent) direction as the central target fish. The presentation of the fish was preceded by one of four cue conditions: no cue, centre cue, double cue, or spatial cue. The cues gave information on when (alerting) or where (orienting) an upcoming target would appear. The task included one practice block with 24 trials; one block with only targets (50% left, 50% right) and the other with targets and flankers (50% congruent, 50% incongruent), followed by three experimental blocks with 48 trials in each. Accuracy, reaction time (RT) and omission errors (failure to respond) were recorded. Any RTs < 100 ms were excluded from the data set prior to calculation of the mean RTs^64^. Three subtractions were computed to obtain alerting (RT no cue—RT double cue, excluding incongruent trials), orienting (RT centre cue—RT spatial cue, excluding incongruent trials) and conflict scores (RT incongruent—RT congruent^65^). The main variable of interest is the conflict score, with higher scores indicating greater deficits in this attention network. Test–retest reliability for scores of each attentional network provided by the ANT are high, with scores on the conflict network being the most reliable (0.77)^66^.

*Working Memory (Verbal)* was assessed using a computer-based Auditory Digit Span Test—Backwards^67,68^. In each trial an auditory sequence of numbers was presented, beginning with a span of two numbers. Students were required to retain the numbers and recall them in the reverse order (i.e., recalling the last digit first). Depending on the accuracy of responses to trials in the previous span, the number sequence was increased by one number, or the task was ended (if two trials of the same span length were answered incorrectly). The longest sequence correctly recalled (digit span) and the total number of correctly recalled sequences was recorded. These variables were multiplied to provide a total score which is the main variable of interest for this measure. Higher scores indicate better verbal working memory performance. Computer-based measures of digit span are reported to have increased reliability and precision compared to traditional delivery, with test–retest reliabilities ranging from 0.64 to 0.84^67^.

*Working Memory (Visuo-spatial)* was assessed using the Corsi Block Tapping Task^69^. In this task students were presented with nine squares randomly arranged on the computer screen. Each trial consisted of a sequence of blocks that lit up in a predetermined order (1000 ms per block). Students were required to remember and reproduce the sequence by selecting the same blocks following the same sequence. Sequence length commenced at two blocks and following two consecutive correct responses of the same sequence length, increased by one block, up to a total of nine blocks. The task was discontinued after two trials of the same length were incorrectly recalled or when the child completed all trials in the task. The main variable of interest is the total score, which is the product of the block span and total number of correctly recalled sequences. Higher scores indicate better visuospatial working memory performance. The computerised version of the Corsi task has been shown to be a valid measure across multiple age groups^70,71^, and has good test–retest reliability (0.75)^72^.

*Cognitive Flexibility (Switching)* was assessed using a computerised version of the Modified Wisconsin Card Sorting Test [WCST; Ref.^73^]. Students were asked to sort 48 non-ambiguous cards into four different categories based on either colour, shape, or number (i.e., sorting criteria). The student was not made aware of the sorting criteria but was provided with corrective feedback after each trial to assist in determining the correct sorting criteria. After 6 consecutive correct trials, the sorting criteria changed. This task recorded proportion of perseveration errors (i.e., proportion of incorrect matches which correspond to a previous sorting criterion after a new criterion has been introduced, relative to total trials), non-perseveration errors (i.e., proportion of incorrect matches that do not reflect the previous or the current sorting rule), and proportion of total errors. Proportion of total errors is the primary outcome variable for this measure. The WCST has excellent reliability (≥ 0.90; Ref.^74^), is a valid measure of perseveration errors and categorising efficiency for children 4–13 years old^75^ and has been used extensively with children^76^.

*Cognitive Flexibility (Matching)* was assessed using the Dimensional Change Card Sort test [DCCS; Ref.^57^. In this task, students were required to sort picture cards (e.g., boats and rabbits), according to one dimension (e.g., colour) and then another dimension (e.g., shape). Students completed six trials before the sorting rule changed (the ‘pre-switch’ phase), and six trials after the sorting rule changed (the ‘post-switch’ phase). Students were explicitly told which sorting rule to apply for each trial in the pre- and post-switch phases to reduce the demands on memory. If students sorted at least five out of six post-switch trials correctly, they passed the ‘post-switch’ phase and moved onto the ‘advanced’ stage. In the ‘advanced’ phase the sorting rule changed on a trial-by-trial basis. Students were informed of this change by a verbal cue (the word ‘colour’ or ‘shape’). Students passed the ‘advanced’ phase if they sorted at least nine out of twelve trials correctly. The main variable of interest for this task is total correct ‘advanced’ phase responses (0–12). The secondary outcome variable is a score of 0–3 based on students passing or failing various phases: scores of 0 are assigned if students failed the ‘pre-switch’ phase, scores of 1 are assigned if students passed the ‘pre-switch’ phase only, scores of 2 are assigned if students passed the ‘post-switch’ phase but not the ‘advanced’ phase, and scores of 3 are assigned if students passed the ‘advanced’ phase. The DCCS has been reported to have excellent convergent validity^77^ and excellent test–retest reliability in children with an intra-class correlation of 0.90–0.94^78^.

### Secondary outcome measures|Far transfer

*Executive Dysfunction* in the home environment was assessed using the parent version of the Behavior Rating Inventory of Executive Function, Second Edition (BRIEF-2^79^). The BRIEF-2 consists of 63 items across nine sub-domains: Inhibit, Self-Monitor, Shift, Emotional Control, Initiate, Working Memory, Plan/Organize, Task-Monitor and Organization of Materials. Parents rated students’ behaviour as occurring “often”, “sometimes” or “never” over the past month. Three broad indices were calculated: Behavior Regulation (BRI), Emotion Regulation (ERI) and Cognitive Regulation (CRI), as well as an overarching summary score: Global Executive Composite (GEC). For all subdomains and indices, higher scores indicate greater executive dysfunction with *T* scores from 60 to 64 considered mildly elevated, 65 to 69 potentially clinically elevated and those above 70 clinically elevated. The main variable of interest for this measure is the GEC. The BRIEF-2 has high internal consistency with index and composite scores ranging from 0.76 to 0.97, high test–retest reliability (GEC = 0.88), and good internal validity (0.41 to 0.83; Ref.^80^).

*Social and Emotional Wellbeing* was assessed using the Strengths and Difficulties Questionnaire [SDQ; Ref.^81^] completed by both parents and educators of participating students. The SDQ is suitable for students 3 to 16 years of age and consists of 25 items divided between five subscales: Emotional Symptoms, Conduct Problems, Hyperactivity/Inattention, Peer Relationship Problems and Pro-Social Behaviour. Parents and educators rated items as “not true”, “somewhat true” or “certainly true” over the past month. A total difficulties score was calculated by summing four deficit-focused subscales (i.e., excluding prosocial behaviour). Higher scores indicate greater social/emotional problems. The SDQ has good reliability (0.67 to 0.90), and high criterion and predictive validity in children^82^.

### Feasibility measures

*Feasibility* of the intervention was assessed through a 25-item educator survey that assesses 6 dimensions of feasibility that are relevant for intervention evaluation^83^. These dimensions include acceptability, practicality, integration, adaptability, implementation, and effectiveness. Educators were asked to rate statements about the intervention on a 5-point Likert scale from “Completely Disagree” to “Completely Agree”, and were given the option to provide additional comments at the end of the survey. Scores ≥ 3 indicate that the program is deemed feasible and acceptable (see Supplementary Information for a full list of items).

*Perceived effectiveness* was assessed through a brief 6 item parent survey. Parents were asked on a scale of 1–5 whether they have noticed a change in their child across the following areas: cognitive skills (impulsivity, memory, flexibility, and attention), social competence, emotion regulation, and mental health/wellbeing. Higher scores indicate better perceived effectiveness.

**Table 2:** Schedule of measures.

	Measure	Main variable	Duration	Timepoints		
				T1	T2	T3
Students						
General cognition	KBIT-2	FSIQ	15 min	X		
Inhibitory control						
Response inhibition	OSARI	SSRT	20 min	X	X	X
Interference control	Child-ANT	Conflict score	25 min	X	X	X
Working memory						
Verbal	Digit span	Total score	5 min	X	X	X
Visuospatial	Corsi block tapping	Total score	5 min	X	X	X
Cognitive flexibility						
Switching	WCST	Proportion of total errors	5 min	X	X	X
Matching	DCCS	Total correct responses on advanced trials	5 min	X	X	X
Parents/caregivers						
Intrinsic motivation	DMQ	Total cognitive persistence subscale score	5 min	X		
Family functioning	FAD	Total score	5 min	X		
Everyday EF	BRIEF-2	GEC score	10 min	X	X	X
Social/emotional wellbeing	SDQ	Total SDQ score	5 min	X	X	X
Effectiveness	Online survey	Total score	5 min		X	
Educators						
Social/emotional wellbeing	SDQ	Total SDQ score	5 min	X	X	X
Feasibility	Online survey	Total score			X	

A summary of all measures, main outcome variables, measure duration and timepoint.

*T1* pre-intervention, *T2* post-intervention, *T3* 3-month follow-up, *KBIT-2* Kaufmann Brief Intelligence Test Second Edition, *FSIQ* Full scale intelligence quotient, *OSARI* Open-Source Anticipatory Response Inhibition Task, *SSRT* Stop Signal Reaction Time, *ANT* Attention Network Task, *WCST* Wisconsin Card Sorting Task, *DCCS* Dimensional Change Card Sort, *DMQ* Dimensional Mastery Questionnaire, *FAD* Family Assessment Device, *EF* Executive Functioning, *BRIEF* Behavior Rating Inventory of Executive Function, *GEC* Global Executive Composite, *SDQ* Strengths and Difficulties Questionnaire.

### Sampling plan

The R package pwr^84^ was used to calculate an a priori power analysis for the analysis plan outlined below. The effect size for the power calculation was based on previous RCTs assessing executive functioning training in young children which reported medium (*n* = 47; g = 0.4–0.5^85^) to large effect sizes (*n* = 87; g = 0.7^86^) for our near transfer outcome measures (inhibitory control, working memory and cognitive flexibility). To yield a power of 95%, assuming equal group sizes, a total sample of 210 students (105 students per group) was required to detect a medium effect size and a total sample of 84 (42 students per group) was required to detect a large effect size. Given this is a school-based trial, all pre-intervention assessments, the 7-week intervention and post-intervention assessments needed to occur within the same school term (~ 10 weeks). Due to these practical restrictions, alongside COVID-19 restrictions at the time of the trial which prevented large scale research trials being conducted through schools, a maximum feasible sample of 140 was expected. This sample would provide 83.6% power to detect a medium effect size and 99.7% power to detect a large effect size on our outcome measures. Recruitment was stopped as soon as the maximum feasible sample size threshold was met.

To ensure the sample was naturalistic, students were only excluded from the trial if they had (a) an intellectual disability (FSIQ < 70; confirmed by the K-BIT2), or (b) significant motor, communication, or sensory impairments (reported by parents) that would impact their ability to fulfil the requirements of the trial. Although students were not excluded if they were taking medication, parents were requested to keep the medication dosage unchanged between the date of enrolment and the follow-up session. Students who performed poorly on pre-intervention assessment practice blocks were excluded from the sampling procedure and all analyses. Participants were withdrawn if they: (a) violated study protocol; (b) experienced a serious or intolerable adverse event; or (c) experienced a decline in wellbeing as reported by classroom educators.

### Analysis plan

Efficacy of the intervention was assessed on an intent-to-treat approach where all trial data was analysed regardless of compliance to the intervention. Analyses of change in the primary (response inhibition) and secondary outcome measures (interference control, working memory, cognitive flexibility, everyday executive functioning, and social/emotional wellbeing) between pre-intervention to post-intervention (short term effects) and pre-intervention to follow-up (sustained effects) as a function of group (intervention *vs* teaching as usual) were conducted using Bayesian latent change score models^87^. Bayesian latent change score models enable direct comparison of treatment effects between randomised groups, as well as quantification of variance in response to treatment within groups, such that individual differences in treatment response can be evaluated^88^. The continuous outcome measures were specified as single-indicator latent variables (i.e., factors) to remove measurement error, increase statistical power, and enable estimation in small samples^87^. Bayes factors were used to quantify the strength of evidence for the H_0_ versus the H_1_ model^89,90^. Bayesian estimation overcomes issues of small sample size, missing data and Type I error^91,92^. Although participants were randomised at an individual level, intra-cluster correlation coefficients at each time point were reported to provide some indication of the relative variability between and within classrooms.

The pre-intervention value of the outcome measure of interest, IQ (scores on the Kaufman Brief Intelligence Test—2nd Edition) and age were statistically controlled for as covariates in the models. Mean values and all primary and secondary outcomes were tabulated by group and time point. For all models, the point estimate of the effect of interest and its 95% Confidence Interval (CI) will be provided. Given the trial has multiple outcomes and that these outcomes may be correlated with each other, the family wise error rate (FWER) was controlled statistically^69^. A sensitivity analysis was conducted to assess whether intervention outcomes differed for those who did (compliers: ≥ 16 training sessions) and did not (non-compliers: < 16 training sessions) adhere to the required intervention schedule. Logistic regression models with demographic factors (gender identity, general cognition, motivation, SES, and family functioning) were then conducted to assess whether those factors influenced which students did not comply with the intervention.

Management of missing data was determined based on the amount and pattern of missing data. Less than 20% data missing at random across all time points indicates good retention and low concern for study validity^70^. Full-information estimation techniques were used to handle missing data, as listwise deletion can lead to biased estimates and loss of power^93^. Specifically, multiple imputation with demographic variables serving as auxiliary variables were utilised to generate unbiased estimates of the missing data^94,95^. Multiple imputation was implemented using the Multivariate Imputation by Chained Equations (MICE) package in R^96^. Bayesian structural equation modelling enabled unbiased estimates with missing data under the assumptions of Missing At Random (MAR)^95^. Restrictions of range was examined through exploration of the data distributions and any ceiling/floor effects were handled using Thorndike’s Case 2 formula^97^.

## Results

**Figure 2:**
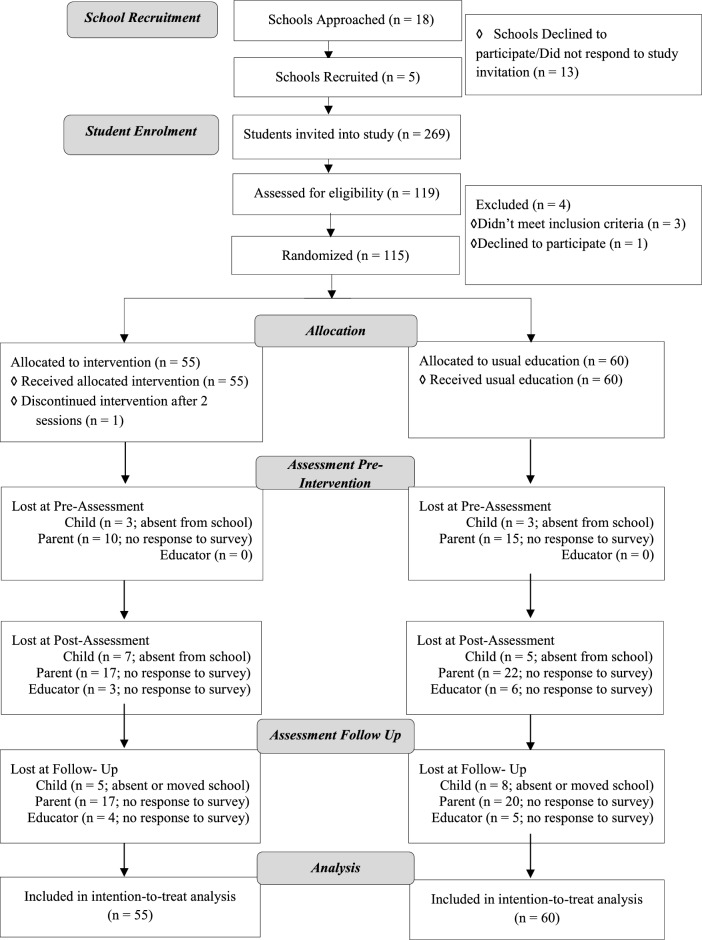
CONSORT flow diagram.

Of the 119 students assessed for eligibility, a total of 115 students aged 6 to 8 years (M = 7.46; SD = 0.61) across 5 remote schools in Victoria took part in the study. Similar numbers of children were randomly allocated intervention (*n* = 55) and control (*n* = 60) groups (see Fig. 2 for CONSORT flow diagram). As shown in Table 3, children’s general cognitive ability, their family characteristics, and intrinsic motivation at pre-intervention were similar across groups (all p > 0.05). The majority of the outcome variables used in the analyses had < 20% missing data across all time points with missing data occurring completely at random. There were two exceptions: parent/caregiver-rated EF (measured by the BRIEF-2) had 26.7% to 37.9% data missing across each timepoint due to low response rates, and response inhibition (measured by the OSARI) had 33.9% to 55.7% data missing across each timepoint due to technical issues with open-source software that led to the corruption of participant data. A missing values analysis was conducted which found that data were missing completely at random, irrespective of the amount of data missing. Hence, as indicated in the Methods section, demographic variables were used to impute missing data via the Multivariate Imputation by Chained Equations (MICE) package in R. This was done once, and the resulting data were used in analyses.

**Table 3:** Pre-intervention child and family characteristics.

	Control	Intervention	Overall	Missing
	(N = 60)	(N = 55)	(N = 115)	N (%)
Male (%)	36 (60)	27 (49)	63 (55)	
Age (years)				
Mean (SD)	7.48 (0.66)	7.43 (0.56)	7.46 (0.61)	
Median [Min, Max]	7.56 [6.32, 8.86]	7.45 [6.55, 8.32]	7.50 [6.32, 8.86]	
Full scale IQ (KBIT-2)				2 (1.74)
Mean (SD)	104.81 (14.10)	104.69 (14.48)	104.75 (14.22)	
Median [Min, Max]	105.5 [74, 131]	105 [70, 135]	105 [70, 135]	
Caregiver-reported diagnosis				24 (20.87)
Autism spectrum disorder (%)	1 (1.67)	–	1 (0.87)	
ADHD (%)	7 (11.67)	1 (1.82)	8 (6.96)	
Dyslexia (%)	1 (1.67)	–	1 (0.87)	
ODD (%)	–	1 (1.82)	1 (0.87)	
Developmental delay (%)	1 (1.67)	–	1 (0.87)	
Specific learning disorder (%)	1 (1.67)	–	1 (0.87)	
Family functioning (FAD)				29 (25.22)
Mean (SD)	1.49 (0.43)	1.40 (0.31)	1.44 (0.38)	
Median [Min, Max]	1.33 [1,2.5]	1.4 [1,2.33]	1.37 [1, 2.5]	
Intrinsic motivation (DMQ)				27 (23.49)
Mean (SD)	3.24 (0.89)	3.29 (0.89)	3.26 (0.89)	
Median [Min, Max]	3.17 [1.5, 4.67]	3.33 [1.67,5]	3.17 [1.5, 5]	
Highest caregiver education (n)				24 (20.87)
Year 11 or below (%)	5 (8.33)	5 (9.09)	10 (8.70)	
Year 12 (%)	4 (6.66)	2 (3.64)	6 (5.22)	
Certificate (%)	3 (5.0)	11 (20.0)	14 (12.17)	
Partial higher education degree (%)	5 (8.33)	4 (7.27)	9 (7.83)	
Completed higher education degree (%)	22 (36.67)	19 (35.55)	41 (35.65)	
Postgraduate degree (%)	6 (10)	5 (9.09)	11 (9.57)	
Employment status				24 (20.87)
Employed^a^ (%)	38 (63.33)	37 (67.27)	75 (65.22)	
Full time student (%)	1 (1.66)	1 (1.82)	2 (1.74)	
Full time parent/carer (%)	4 (6.67)	6 (10.91)	10 (8.70)	
Not in paid employment (%)	2 (3.33)	2 (3.64)	4 (3.48)	
Child’s cultural background (n)				33 (28.70)
Aboriginal/Torres Strait Islander (%)	5 (8.33)	5 (9.09)	10 (8.70)	
Asian (%)	3 (5.00)	3 (5.45)	6 (5.22)	
White/European (%)	30 (50)	30 (54.55)	60 (52.17)	
African & Middle Eastern (%)	2 (3.33)	–	2 (1.74)	
Other (%)	2 (3.33)	2 (3.64)	4 (3.48)	
Guardian’s relationship to child (n)				24 (20.87)
Mother (%)	48 (80.0)	47 (85.45)	95 (82.61)	
Father (%)	8 (13.33)	7 (12.73)	15 (13.04)	
Other family member (%)	2 (3.33)	1 (1.82)	3 (2.61)	
Step/Foster parent (%)	2 (3.33)	–	2 (1.74)	

*KBIT-2* Kaufmann Brief Intelligence Test Second Edition, *IQ* Intelligence Quotient, *ADHD* Attention Deficit Hyperactivity Disorder, *ODD* Oppositional Defiant Disorder, *FAD* Family Assessment Device, *DMQ* Dimensional Mastery Questionnaire.

^a^Includes fulltime, part-time and casual employment.

### Intervention effects

Mean scores and standard deviations for primary and secondary outcome measures at each timepoint are presented in Table 4. Latent change score models^87^ were used to estimate within and between group variance at once as well as sequential effects of variables on each other over time and an unobserved latent change score (see the Supplementary Information for detailed descriptions of the models implemented).

**Table 4:** Mean values and standard deviations for all outcome measures per treatment group at each assessment time point.

Outcome measure	Group	Pre-intervention (T1)			Post-intervention (T2)			Follow-up (T3)			Pre-to-Post (T1-T2)		Pre-to-FU (T1-T3)	
		*n*	*M*	SD	*n*	*M*	SD	*n*	*M*	SD	Diff	95% CI	Diff	95% CI
Inhibitory control														
Response inhibition *(OSARI)*	Control	39	369.51	121.51	36	356.40	121.16	21	407.92	163.59	1.98	[− 54.79, 58.74]	25.58	[− 117.78, 168.93]
	Intervention	37	376.10	125.59	29	352.08	110.64	30	369.91	122.92	− 28.94	[− 92.05, 34.17]	− 2.36	[− 78.20, 73.48]
Interference control (*Child − ANT)*	Control	57	122.43	101.64	54	109.31	88.31	51	98.85	84.62	− 12.05	[− 49.16, 25.06]	− 23.05	[− 55.67, 9.57]
	Intervention	52	93.48	125.75	46	82.36	67.45	50	99.56	85.62	− 11.83	[− 57.87, 34.22]	4.22	[− 38.93, 47.37]
Working memory														
Verbal *(Digit Span)*	Control	57	12.14	8.60	54	15.09	9.32	49	12.29	6.26	2.77	[0.05, 5.49]	0	[− 2.54, 2.54]
	Intervention	52	10.90	8.68	48	13.69	8.19	50	13.76	8.71	3.16*	[0.27, 6.05]	3.42*	[0.73, 6.10]
Visuospatial *(Corsi Block Tapping)*	Control	57	18.56	13.65	55	25.11	13.65	52	22.15	13.22	6.54**	[2.20, 10.87]	4.12	[− 0.40, 8.63]
	Intervention	52	18.48	13.36	48	24.79	14.07	50	22.08	15.52	7.69**	[2.87, 12.51]	3.02	[− 1.33, 7.37]
Cognitive flexibility														
Switching *(WCST)*	Control	57	0.57	0.16	54	0.44	0.16	52	0.45	0.19	− 0.12***	[0.06, 0.17]	− 0.11***	[0.06, 0.16]
	Intervention	51	0.53	0.16	47	0.37	0.19	50	0.40	0.18	− 0.16***	[0.10, 0.23]	− 0.12***	[0.06, 0.18]
Matching *(DCCS)*	Control	57	8.60	2.86	54	9.85	2.44	49	9.96	2.16	1.25***	[0.53, 1.96]	1.25**	[0.44, 2.06]
	Intervention	52	8.10	2.76	48	8.69	2.83	50	9.60	2.30	0.27	[− 0.71, 1.24]	1.54***	[0.83, 2.25]
Everyday EF *(BRIEF − 2)*	Control	42	53.45	12.52	36	52.64	11.54	38	53.97	12.94	− 1.09	[− 3.07, 0.88]	0.97	[− 0.92, 2.86]
	Intervention	43	49.44	9.01	36	52.11	7.60	36	53.36	9.71	1.88**	[0.69, 3.06]	1.76*	[0.31, 3.22]
Social/emotional wellbeing														
Caregiver *(SDQ)*	Control	45	10.44	6.50	38	8.63	5.58	40	8.78	5.86	− 1.83**	[− 3.10, − 0.55]	− 0.97	[− 2.41, 0.46]
	Intervention	45	8.73	4.94	38	9.11	5.26	38	9.08	5.87	0.59	[− 0.64, 1.82]	0.19	[− 1.11, 1.50]
Educators *(SDQ)*	Control	60	7.63	5.24	54	8.15	5.40	55	8.73	6.96	0.43	[− 0.39, 1.24]	1.25*	[0.18, 2.33]
	Intervention	55	6.80	5.30	52	7.42	6.37	51	7.18	5.91	0.56	[− 0.32, 1.43]	0.84	[− 0.17, 1.86]

All presented scores are raw scores. *p < .05, **p < .01, ***p < 001.

*FU* Follow Up, *M* Mean, SD Standard Deviation, *Diff*. between group difference in the change over time, *CI* confidence interval, *OSARI* Open-Source Anticipatory Response Inhibition Task, *ANT* Attention Network Task, *WCST* Wisconsin Card Sorting Task, *DCCS* Dimensional Change Card Sort, *BRIEF* Behavior Rating Inventory of Executive Function, *SDQ* Strengths and Difficulties Questionnaire.

### Near transfer|Inhibitory control

No significant improvements in response inhibition or interference control performance were observed from pre-to-post-intervention, or pre-intervention to follow-up for either group (see Table 4). Tables 5 and 6 present the fixed and random effects for all models for the pre-post comparisons and pre-FU comparisons, respectively. Both tables show poor model fit across all increasingly invariant models, with posterior predictive p-value (PPP) not close to 0.5 for any model, and most < 0.05, indicating extremely poor fit. Most model differences were negligible, with the exception of Model 3 for the pre-to-post comparisons, which was superior to Model 2. However, none of the regressions within the model were significant. In addition, the Leave-One-Out Information Criterion (LOOIC) difference score had a large standard error, suggesting that there was great uncertainty around this estimated difference in model fit. Together these findings suggest that there were no significant changes in inhibitory control performance for either group from pre-to-post-intervention, or pre-intervention to follow-up, nor any difference in the degree of change across groups over time.

**Table 5:** Model outcome for pre-to-post intervention comparisons across increasingly relaxed measurement invariance for Near (response inhibition) and Far transfer outcome measures.

	Model 1: Invariant over groups *M* (SD) [CI]		Model 2: Free latent change intercepts *M* (SD) [CI]		Model 3: Free crossed and lagged regressions *M* (SD) [CI]		Model 4: Free latent change regression *M* (SD) [CI]	
	C	I	C	I	C	I	C	I
(a) Fixed parameters (intercepts and simultaneous regression coefficients)								
1 → ∆Near	**2.26 (0.99) [0.29–4.18]**	=	**2.39 (0.99) [0.59–4.39]**	1.85 (0.99) [− 0.04–3.81]	**2.34 (0.98) [0.42–4.17]**	**2.21 (1.01) [0.16–4.04]**	1.69 (0.95) [− 0.07–3.72]	1.56 (0.93) [− 0.19–3.42]
Near[0] → ∆ Near	− 0.88 (3.74) [− 9.43–8.45]	=	− 0.79 (3.60) [− 8.54–8.26]	=	− 0.54 (8.22) [− 17.32–16.43]	− 0.15 (8.14) [− 17.13 16.70]	− 0.003 (7.23) [− 15.48–14.39]	− 0.37 (8.30) [− 16.73–15.55]
Far[0] → ∆Near	− 0.22 (0.20) [− 0.64–0.15]	=	− 0.25 (0.21) [− 0.67–0.16]	=	− 0.39 (0.25) [− 0.90–0.09]	0.30 (0.45) [− 0.55–1.20]	0.33 (0.24) [− 0.83–0.08]	0.29 (0.40) [− 0.49–1.06]
(b) Fixed parameters (intercepts and simultaneous regression coefficients)								
1 → ∆Far	− 0.09 (1.75) [− 3.69–3.50]	=	− 0.16 (1.76) [− 3.69–3.49]	0.14 (1.46) [− 2.92–3.20]	− 0.07 (1.75) [− 3.65–3.62]	0.25 (1.58) [− 2.91–3.70]	0.50 (2.60) [− 5.72–5.73]	0.13 (2.33) [− 4.62–5.28]
Near[0] → ∆Far	− 0.10 (3.57) [− 8.67–7.28]	=	0.12 (2.95) [− 6.19–7.46]	=	0.41 (7.54) [− 7.42–14.83]	0.07 (6.78) [− 14.66–13.65]	0.16 (8.10) [− 16.42–17.00]	0.15 (7.85) [− 16.14–15.82]
Far[0] →∆Far	− 0.05 (0.24) [− 0.54–0.43]	=	− 0.04 (0.23) [− 0.50–0.46]	=	− 0.15 (0.30) [− 0.78–0.49]	0.24 (0.40) [− 0.48–1.13]	0.20 (0.62) [− 1.71–1.10]	0.20 (0.82) [− 1.39–1.74]
∆Near → ∆Far	− 0.06 (0.77) [− 1.67–1.56]	=	− 0.004 (0.72) [− 1.51–1.50]	=	− 0.06 (0.66) [− 1.45–1.31]	=	− 0.18 (1.86) [− 4.15–3.31]	− 0.01 (1.61) [− 3.25–3.50]
(c) Random parameters (variance)								
∆ Near	0.16 (0.27)	=	0.16 (0.30)	0.26 (0.44)	0.09 (0.14)	0.14 (0.24)	0.06 (0.11)	0.11 (0.20)
∆ Far	0.05 (0.10)	=	0.05 (0.08)	0.04 (0.07)	0.04 (0.08)	0.03 (0.09)	0.06 (0.14)	0.06 (0.28)
(d) Factor loadings								
Near								
ANT	1	=	1	=	1	=	1	=
OSARI	− 0.18 (0.24)	=	− 0.14 (0.17)	=	− 0.20 (0.21)	=	− 0.38 (0.28)	=
Far								
GEC	1	=	1	=	1	=	1	=
SDQ	0.78 (0.11)	=	0.80 (0.12)	=	0.82 (0.12)	=	0.79 (0.11)	=
(e) Model fit and comparisons								
PPP/LOOIC	0.019/237.52		0.033/3237.76		0.008/3228.04		0.005/3224.42	
LOO diff (SE)	=		** − 0.12 (0.36)**		**4.18 (5.70)**		** − 1.78 (1.62)**	

Significant values are in bold.

PPP close to 0.5 indicates good model fit.

*C* Control Group, *I* Intervention Group, *M* Mean, *SD* Standard deviation, *CI* credible interval, *ANT* Child Attention Network Task, *OSARI* Open-Source Anticipatory Response Inhibition Task, *GEC* Global Executive Composite, *SDQ* mean teacher and parental rating on the Strengths and Difficulties Questionnaire, *PPP* Posterior Predictive P-value, *LOOIC* Leave-One-Out Information Criterion, *LOO diff* difference in Leave One Out, *SE* standard error.

**Table 6:** Model outcome for pre-intervention to follow-up comparisons across increasingly relaxed measurement invariance for Near (response inhibition) and Far transfer outcome measures.

	Model 1: Invariant over groups *M* (SD) [CI]		Model 2: Free latent change intercepts *M* (SD) [CI]		Model 3: Free crossed and lagged regressions *M* (SD) [CI]		Model 4: Free latent change regression *M* (SD) [CI]	
	C	I	C	I	C	I	C	I
(a) Fixed parameters (intercepts and simultaneous regression coefficients)								
1→ ∆Near	**1.85 (0.99) [0.14–3.88]**	=	**2.15 (1.02) [0.19–4.19]**	2.08 (1.01) [− 0.20–4.17]	**2.06 (1.01) [0.08–4.12]**	1.70 (1.00) [− 0.16–3.68]	1.69 (0.95) [− 0.07–3.72]	1.55 (0.98) [− 0.24–3.53]
Near[0] →∆ Near	− 0.11 (5.86) [− 12.58–12.89]	=	− 0.30 (5.40) [− 12.45–12.16]	=	− 0.10 (8.38) [− 16.57–17.65]	− 0.15 (8.44) [− 16.62–16.21]	0.23 (7.46) [− 15.01–15.62]	− 0.24 (8.60) [− 18.11–16.73]
Far[0] →∆Near	0.03 (0.19) [− 0.35–0.40)	=	0.03 (0.21) [− 0.41–0.44]	=	0.09 (0.26) [− 0.43–0.61]	− 0.18 (0.45) [− 1.07–0.72]	0.09 (0.24) [− 0.39–0.57]	− 0.15 (0.41) [− 1.03–0.67]
(b) Fixed parameters (intercepts and simultaneous regression coefficients)								
1 → ∆Far	− 0.02 (1.64) [− 3.39–3.48]	=	− 0.16 (1.66) [− 3.51–3.59]	0.02 (1.78) [− 3.40–3.64]	0.02 (1.63) [− 3.09–3.40]	0.10 (1.53) [− 3.05–3.35]	0.50 (2.60) [− 5.72–5.73]	− 0.30 (2.26) [− 5.10–4.54]
Near[0] → ∆Far	− 0.34 (5.69) [− 12.34–12.89]	=	0.32 (5.16) [− 11.23–12.39]	=	0.30 (7.02) [− 14.48–14.98]	0.14 (7.66) [− 15.88–16.10]	0.41 (8.39) [− 17.30–17.17]	0.45 (8.41) [− 16.88–17.20]
Far[0] →∆Far	0.09 (0.16) [− 0.23–1.00]	=	**0.10 (0.19) [0.27–0.53]**	=	0.05 (0.36) [− 0.60–0.80]	0.03 (0.38) [− 0.74–0.77]	0.17 (0.44) [− 0.68–1.13]	0.09 (0.65) [− 1.22–1.56]
∆Near → ∆Far	− 0.06 (0.89) [− 1.96–1.60]	=	− 0.004 (0.77) [− 1.67–1.54]	=	− 0.03 (0.74) [− 1.70–1.24]	=	− 0.44 (1.84) [− 4.00–3.17]	0.22 (1.67) [− 3.43–3.66]
(c) Random parameters (variance)								
∆ Near	0.14 (0.22)	=	0.20 (0.29)	0.21 (0.36)	0.24 (0.35)	0.19 (0.31)	0.11 (0.18)	0.16 (0.27)
∆ Far	0.11 (0.14)	=	0.11 (0.15)	0.12 (0.65)	0.06 (0.12)	0.06 (0.12)	0.12 (0.26)	0.07 (0.22)
(d) Factor loadings								
Near								
ANT	1	=	1	=	1	=	1	=
OSARI	− 0.19 (0.46)	=	− 0.12 (0.28)	=	− 0.13 (0.27)	=	− 0.20 (0.44)	=
Far								
GEC	1	=	1	=	1	=	1	=
SDQ	0.85 (0.10)	=	0.86 (0.10)	=	0.86 (0.10)	=	0.86 (0.10)	=
(e) Model fit and comparisons								
PPP/LOOIC	0.023/3274.93		0.045/3276.51		−0.010/3277.62		0.014/3275.70	
LOO diff (SE)	=		− 0.77 (1.82)		1.06 (1.64)		− 1.45 (1.76)	

PPP close to 0.5 indicates good model fit.

Significant values are in bold.

*C* Control Group, *I* Intervention Group, *M* Mean, *SD* Standard deviation, *CI* credible interval, *ANT* Child Attention Network Task, *OSARI* Open-Source Anticipatory Response Inhibition Task, *GEC* Global Executive Composite, *SDQ* mean teacher and parental rating on the Strengths and Difficulties Questionnaire, *PPP* Posterior Predictive P-value, *LOOIC* Leave-One-Out Information Criterion, *LOO diff* difference in Leave One Out, *SE* standard error.

### Secondary outcome measures

#### Near transfer|Working memory

Significant improvements in verbal working memory performance were observed for the intervention group from pre-to-post intervention (p < 0.05) and pre-intervention to 3-month follow-up (p < 0.05; see Table 4). Both groups experienced significant improvements in visuospatial working memory performance from pre-to-post intervention (*p* < 0.01). However, the latent change score models investigating improvements in working memory (see Tables 7 and 8) showed very poor fit (PPP < 0.05), and small differences between models (LOOIC difference < 2) indicated that students in the intervention group did not experience greater change in working memory performance at any timepoint compared to the control group.

**Table 7:** Model outcome for pre-to-post intervention comparisons across increasingly relaxed measurement invariance for Near (working memory) and Far transfer outcome measures.

	Model 1: Invariant over groups *M* (SD) [CI]		Model 2: Free latent change intercepts *M* (SD) [CI]		Model 3: Free crossed and lagged regressions *M* (SD) [CI]		Model 4: Free latent change regression *M* (SD) [CI]	
	C	I	C	I	C	I	C	I
(a) Fixed parameters (intercepts and simultaneous regression coefficients)								
1 → ∆Near	0.19 (0.86) [− 1.50–1.92]	=	0.18 (0.92) [− 1.71–2.02]	0.11 (0.90) [− 1.63–1.95]	0.19 (0.88) [− 1.54–1.92]	0.13 (0.90) [− 1.53–1.89]	0.16 (0.87) [− 1.55–1.86]	0.11 (0.86) [− 1.67–1.77]
Near[0→ ∆Near	− 0.38 (0.20) [− 0.75–0.02)	=	− 0.40 (0.19) [− 0.75–0.03]	=	− 0.24 (0.32) [− 0.82–0.45]	− 0.40 (0.27) [− 0.87–0.18]	− 0.23 (0.34) [− 0.86–0.42]	− 0.38 (0.28) [− 0.88–0.20]
Far[0] → ∆Near	− 0.19 (0.12) [− 0.45–0.05]	=	− 0.19 (0.12) [− 0.44–0.04]	=	− 0.18 (0.14) [− 0.46–0.08]	− 0.27 (0.26) [− 0.74–0.25]	− 0.18 (0.14) [− 0.47–0.09]	− 0.27 (0.25) [− 0.80–0.21]
(b) Fixed parameters (intercepts and simultaneous regression coefficients)								
1 → ∆Far	− 0.18 (1.15) [− 2.51–2.17]	=	− 0.22 (1.16) [− 2.53–2.05]	0.10 (1.16) [− 2.01–2.22]	− 0.23 (1.13) [− 2.34–1.91]	0.12 (1.10) [− 2.07–2.39]	− 0.15 (2.18) [− 5.00–4.90]	0.10 (1.86) [− 4.04–4.19]
Near[0] →∆Far	− 0.12 (0.56) [− 1.19–0.69]	=	− 0.05 (0.49) [− 0.79–0.84]	=	0.04 (0.48) [− 0.68–0.94]	− 0.13 (0.52) [− 1.05–0.94]	0.07 (1.48) [− 2.98–2.97]	− 0.22 (1.32) [− 3.29–2.37]
Far[0] → ∆Far	− 0.08 (0.28) [− 0.72–0.37]	=	− 0.05 (0.28) [− 0.50–0.41]	=	− 0.13 (0.27) [− 0.56–0.38]	0.20 (0.46) [− 0.55–1.18]	− 0.13 (0.80) [− 1.88–1.62]	0.14 (1.06) [− 2.14–2.35]
∆Near → ∆Far	0.18 (1.25 [− 3.30–1.91]	=	− 0.02 (1.18) [− 2.12–2.44]		− 0.05 (1.14) [− 2.40–2.40]	=	− 0.01 (3.58) [− 8.50–7.37]	− 0.20 (3.12) [− 7.13–7.45]
(c) Random parameters (variance)								
∆ Near	0.13 (0.17)	=	0.13 (0.17)	0.19 (0.21)	0.12 (0.15)	0.17 (.20)	0.07 (0.12)	0.12 (0.17)
∆ Far	0.06 (0.12)	=	0.04 (0.04)	0.06 (0.15)	0.06 (0.22)	0.04 (0.10)	0.11 (0.50)	0.06 (0.17)
(d) Factor loadings								
Near								
Corsi	1	=	1	=	1	=	1	=
DS	0.39 (0.13)	=	0.38 (0.12)	=	0.39 (0.12)	=	0.40 (0.12)	=
Far								
GEC	1	=	1	=	1	=	1	=
SDQ	0.82 (0.14)	=	0.87 (0.17)	=	0.83 (0.13)	=	0.83 (0.12)	=
(e) Model fit and comparisons								
PPP/LOOIC	0.025/2878.96		0.038/2880.16		0.045/2880.76		0.043/2880.14	
LOO diff (SE)	=		− 0.60 (1.90)		0.30 (2.59)		− 0.31 (0.40)	

PPP close to 0.5 indicates good model fit.

*C* Control Group, *I* Intervention Group, *M* Mean, *SD* Standard deviation, *CI* credible interval, *DS* Digit Span, *GEC* Global Executive Composite, *SDQ* mean teacher and parental rating on the Strengths and Difficulties Questionnaire, *PPP* Posterior Predictive P-value, *LOOIC* Leave-One-Out Information Criterion, *LOO diff* difference in Leave One Out, *SE* standard error.

**Table 8:** Model outcome for pre-intervention to follow-up comparisons across increasingly relaxed measurement invariance for Near (working memory) and Far transfer outcome measures.

	Model 1: Invariant over groups *M* (SD) [CI]		Model 2: Free latent change intercepts *M* (SD) [CI]		Model 3: Free crossed and lagged regressions *M* (SD) [CI]		Model 4: Free latent change regression *M* (SD) [CI]	
	C	I	C	I	C	I	C	I
(a) Fixed parameters (intercepts and simultaneous regression coefficients)								
1 → ∆Near	− 0.11 (0.84) [− 1.75–1.61]	=	− 0.10 (0.89) [− 1.82–1.64]	0.16 (0.88) [− 1.46–1.94]	− 0.07 (0.82) [− 1.66–1.53]	0.14 (0.83) [− 1.47–1.81]	0.15 (0.85) [− 1.51–1.84]	0.10 (0.88) [− 1.63–1.80]
Near[0 → ∆Near	− 0.22 (0.24) [− 0.61–0.29]	=	− 0.17 (0.25) [− 0.59–0.39]	=	− 0.55 (0.84) [− 0.98–0.07]	0.39 (0.41) [− 0.23–1.26]	0.24 (0.50) [− 0.85–0.46]	0.39 (0.29) [− 0.91–0.23]
Far[0] → ∆Near	− 0.12 (0.10) [− 0.30–0.08]	=	− 0.12 (0.10) [− 0.30–0.08]	=	− 0.14 (0.10) [− 0.35–0.05]	− 0.05 (0.23) [− 0.51–0.40]	− 0.18 (0.14) [− 0.46–0.09]	− 0.26 (0.27) [− 0.79–0.26]
(b) Fixed parameters (intercepts and simultaneous regression coefficients)								
1 → ∆Far	− 0.11 (1.42) [− 3.26–2.73]	=	− 0.09 (1.61) [− 3.54–3.12]	− 0.04 (1.56) [− 3.48–3.32]	− 0.09 (1.64) [− 3.40–3.39]	− 0.04 (1.65) [− 3.50–3.33]	− 0.18 (2.36) [− 4.84–4.89]	0.10 (1.85) [− 3.95–3.93]
Near[0] →∆Far	0.05 (0.60) [− 1.10–1.33]	=	0.04 (0.58) [− 1.06–1.28]	=	0.09 (1.43) [− 2.82–2.79]	− 0.10 (1.23) [− 2.77–2.43]	0.15 (1.81) [− 2.87–3.77]	− 0.21 (1.28) [− 3.32–2.55]
Far[0] → ∆Far	0.12 (0.32) [− 0.48–0.76]	=	0.10 (0.33) [− 0.54–0.76]	=	0.13 (0.38) [− 0.70–0.94]	0.07 (0.51) [− 0.90–1.16]	− 0.10 (0.89) [− 1.73–1.73]	0.15 (1.09) [− 2.03–2.60]
∆Near → ∆Far	0.20 (2.02) [− 4.55–4.49]	=	0.20 (2.02) [− 4.32–4.52]	=	0.12 (2.12) [− 4.60–4.54]	=	0.25 (3.87) [− 8.01–9.51]	− 0.30 (2.92) [− 7.31–6.09]
(c) Random parameters (variance)								
∆ Near	0.03 (0.04)	=	0.03 (0.04)	0.09 (0.14)	0.03 (0.04)	0.06 (0.10)	0.06 (0.11)	0.10 (0.18)
∆ Far	0.12 (0.16)	=	0.11 (0.13)	0.08 (0.22)	0.12 (0.21)	0.07 (0.16)	0.09 (0.22)	0.05 (0.21)
(d) Factor loadings								
Near								
Corsi	1	=	1	=	1	=	1	=
DS	0.45 (0.15)	=	0.48 (0.15)	=	0.55 (0.15)	=	0.39 (0.12)	=
Far								
GEC	1	=	1	=	1	=	1	=
SDQ	0.84 (0.09)	=	0.10)	=	0.86 (0.10)	=	0.83 (0.13)	=
(e) Model fit and comparisons								
PPP/LOOIC	0.004/2842.36		0.005/2844.08		0.007/2879.65		.052/2881.39	
LOO diff (SE)	=		− 0.86 (1.89)		− 1.11 (2.06)		− 0.50 (0.30)	

PPP close to 0.5 indicates good model fit.

*C* Control Group, *I* Intervention Group, *M* Mean, *SD* Standard deviation, *CI* credible interval, *DS* Digit Span, *GEC* Global Executive Composite, *SDQ* mean teacher and parental rating on the Strengths and Difficulties Questionnaire, *PPP* Posterior Predictive P-value, *LOOIC* Leave-One-Out Information Criterion, *LOO diff* difference in Leave One Out, *SE* standard error.

#### Near transfer|Cognitive flexibility

Significant improvements in switching performance were observed for both the intervention and control groups from pre-to-post intervention (*p* < 0.001) and from pre-intervention to 3-month follow-up (*p* < 0.001; see Table 4). Significant improvements in matching performance were observed for the control group from pre-to-post intervention (*p* < 0.001) and for both the intervention and control groups from pre-intervention to follow-up (*p* < 0.01). The fixed and random effects for all latent change score models evaluating change in cognitive flexibility are presented in Tables 9 and 10. Although some less invariant models were superior to more invariant models, all models showed poor fit, and none of the parameters in any of the simultaneous regressions in any of the models were significant. Together these findings suggested that the intervention group did not experience greater improvements in cognitive flexibility performance at any timepoint compared to the control group.

**Table 9:** Model outcome for pre-to-post intervention comparisons across increasingly relaxed measurement invariance for Near (cognitive flexibility) and Far transfer outcome measures.

	Model 1: Invariant over groups *M* (SD) [CI]			Model 2: Free latent change intercepts *M* (SD) [CI]		Model 3: Free crossed and lagged regressions *M* (SD) [CI]		Model 4: Free latent change regression *M* (SD) [CI]	
	C	I		C	I	C	I	C	I
(a) Fixed parameters (intercepts and simultaneous regression coefficients)									
1 → ∆Near	− 0.12 (0.67) [− 1.49–1.22]	=		0.12 (0.70) [− 1.29–1.95]	0.07 (0.71) [− 1.56–1.34]	0.15 (0.70) [− 1.61–1.22]	− 0.07 (0.71) [− 1.54–1.29]	− 0.15 (0.64) [− 1.43–1.12]	0.10 (0.78) [− 1.44 (1.70)
Near[0] →∆ Near	− 0.19 (0.18) [− 0.52–0.19)	=		− 0.19 (0.17) [− 0.49–0.18]	=	− 0.27 (0.42) [− 0.67–0.22]	0.12 (0.44) [− 0.46–0.95]	− 0.27 (0.22) [− 0.66–0.21]	− 0.38 (0.28) [− 0.88–0.22]
Far[0] → ∆Near	− 0.06 (0.08) [− 0.10–0.22]	=		0.07 (0.09) [− 0.10–0.24]	=	− 0.07 (0.09) [− 0.12–0.25]	0.10 (0.19) [− 0.26–0.49]	− 0.07 (0.09) [− 0.10–0.24]	− 0.25 (0.26) [− 0.79–0.24]
(b) Fixed parameters (intercepts and simultaneous regression coefficients)									
1 → ∆Far	− 0.18 (1.29) [− 2.78–2.40]	=		− 0.15 (1.46) [− 3.00–3.00]	0.16 (1.36) [− 2.61–2.94]	− 0.15 (1.37) [− 2.73–2.69]	0.15 (1.42) [− 2.55–2.90]	− 0.08 (2.40) [− 4.91–5.51]	0.09 (1.70) [− 3.48–3.68]
Near[0] → ∆Far	− 0.07 (0.55) [− 1.08–0.93]	=		− 0.05 (0.51) [− 1.20–0.93]	=	− 0.08 (0.75) [− 1.64–1.51]	− 0.04 (0.84) [− 1.53–1.57]	0.07 (1.61) [− 3.53–3.50]	− 0.13 (1.34) [− 2.85–2.69]
Far[0] → ∆Far	− 0.05 (0.24) [− 0.52–0.35]	=		− 0.05 (0.23) [− 0.52–0.45]	=	− 0.12 (0.25) [− 0.60–0.38]	0.18 (0.46) [− 0.85–1.02]	− 0.12 (0.55) [− 1.31–1.05]	0.20 (1.04) [− 2.00–2.48]
∆Near → ∆Far	0.24 (2.13) [− 4.64–4.22]	=		− 0.27 (2.07) [− 4.34–4.57]		0.15 (2.08) [− 4.40–4.52]	=	0.16 (4.78) [− 9.56–10.22]	− 0.09 (2.92) [− 6.22–6.71]
(c) Random parameters (variance)									
∆ Near	0.14 (0.17)	=		0.02 (0.04)	0.18 (0.21)	0.02 (0.03)	0.03 (.06)	0.01 (0.02)	0.12 (0.18)
∆ Far	0.06 (0.10)	=		0.05 (0.10)	0.06 (0.16)	0.05 (0.08)	0.04 (0.12)	0.08 (0.27)	0.41 (0.11)
(d) Factor loadings									
Near									
WCST	1	=		1	=	1	=	1	=
DCCS	1.06 (0.39)	=		− 0.99 (0.32)	=	− 1.00 (0.32)	=	− 1.01 (0.33)	=
Far									
GEC	1	=		1	=	1	=	1	=
SDQ	0.79 (0.12)	=		0.83 (0.15)	=	0.82 (0.13)	=	0.81 (0.12)	=
(e) Model fit and comparisons									
PPP/LOOIC	0.003/2810.53			0.013/2805.64		0.015/2808.22		0.018/2817.55	
LOO diff (SE)	=			− 1.95 (2.74)		0.40 (2.71)		** − 4.15 (1.20)**	

PPP close to 0.5 indicates good model fit.

Significant values are in bold.

*C* Control Group, *I* Intervention Group, *M* Mean, *SD* Standard deviation, *CI* credible interval, *WCST* Wisconsin Card Sorting Task, *DCCS* Dimensional Change Card Sort test, *GEC* Global Executive Composite, *SDQ* mean teacher and parental rating on the Strengths and Difficulties Questionnaire, *PPP* Posterior Predictive P-value, *LOOIC* Leave-One-Out Information Criterion, *LOO diff* difference in Leave One Out, *SE* standard error.

**Table 10:** Model outcome for pre-intervention to follow-up comparisons across increasingly relaxed measurement invariance for Near (cognitive flexibility) and Far transfer outcome measures.

	Model 1: Invariant over groups *M* (SD) [CI]		Model 2: Free latent change intercepts *M* (SD) [CI]		Model 3: Free crossed and lagged regressions *M* (SD) [CI]		Model 4: Free latent change regression *M* (SD) [CI]	
	C	I	C	I	C	I	C	I
(a) Fixed parameters (intercepts and simultaneous regression coefficients)								
1 → ∆Near	− 0.16 (0.56) [− 1.30–0.99]	** = **	− 0.19 (0.58) [− 1.37–0.87]	− 0.18 (0.58) [− 1.32–0.93]	0.18 (0.58) [− 1.40–1.00]	− 0.17 (0.59) [− 1.30–1.02]	− 0.15 (0.49) [− 1.26–0.76}	− 0.14 (0.50) [− 1.25–0.76]
Near[0] → ∆ Near	− 0.30 (0.18) [− 0.61–0.09]	** = **	− 0.30 (0.18) [− 0.63–0.09]	=	− 0.23 (0.29) [− 0.75–0.41]	− 0.28 (0.36) [− 0.84–0.37]	0.22 (0.28) [− 0.72–0.39]	− 0.26 (0.46) [− 0.79–0.33]
Far[0] → ∆Near	− 0.14 (0.08) [0.003– − 0.30]	=	0.14 (0.07) [0.01–0.30]	=	0.10 (0.09) [− 0.07–0.29]	0.23 (0.14) [− 0.01–0.52]	0.10 (0.08) [− 0.07–0.27]	0.22 (0.14) [− 0.02–0.51]
(b) Fixed parameters (intercepts and simultaneous regression coefficients)								
1 → ∆Far	− 0.22 (1.36) [− 3.16–2.61]	=	− 0.18 (1.52) [− 3.49–2.92]	− 0.07 (1.50) [− 3.27–2.87]	− 0.18 (1.52) [− 3.34–2.94]	− 0.06 (1.43) [− 3.10–2.98]	− 0.10 (2.13) [− 4.80–4.26]	0.01 (2.02) [− 4.43–4.49]
Near[0] → ∆Far	− 0.38 (0.90) [− 2.17–1.46]	=	− 0.42 (0.87) [− 2.46–1.34}	=	− 0.39 (0.94) [− 2.48–1.47]	− 0.32 (1.12) [− 2.62–1.91]	− 0.46 (1.85) [− 4.68–3.54]	− 0.28 (1.84) [− 4.12–3.58]
Far[0] → ∆Far	0.10 (0.40) [− 0.66–1.01]	=	0.20 (0.41) [− 0.61–1.05]	=	0.19 (0.33) [− 0.53–0.86]	0.16 (0.64) [− 1.26–1.91]	0.19 (0.69) [− 1.30–1.63]	0.09 (1.27) [− 2.50–2.64]
∆Near → ∆Far	− 0.69 (2.67) [− 6.41–5.26]	=	− 0.73 (2.68) [6.61–5.29]	=	− 0.60 (2.59) [− 6.10–4.91]	=	0.58 (5.55) [− 12.53–11.90]	− 0.26 (5.04) [− 10.39–10.77]
(c) Random parameters (variance)								
∆ Near	0.03 (0.04)	=	0.03 (0.04)	0.02 (0.04)	0.03 (0.04)	0.02 (0.04)	0.02 (0.03)	0.01 (0.03)
∆ Far	0.11 (0.15)	=	0.11 (0.28)	0.05 (0.11)	0.12 (0.20)	0.06 (0.10)	0.13 (0.24)	0.07 (0.18)
(d) Factor loading								
Near								
WCST	1	=	1	=	1	=	1	
DCCS	− 1.45 (0.48)	=	− 1.45 (0.51)	=	− 1.43 (0.50)	=	− 1.54 (0.57)	
Far								
GEC	1	=	1	=	1	=	1	
SDQ	0.88 (0.11)	=	0.88 (0.11)	=	0.88 (0.11)	=	0.88 (0.10)	
(e) Model fit and comparisons								
PPP/LOOIC	.004/2842.36		.100/2813.62		.070/2822.93		.090/2817.55	
LOO diff (SE)	=		** − 2.40 (1.21)**		− 1.29 (2.16)		** − 2.69 (1.18)**	

PPP close to 0.5 indicates good model fit.

Significant values are in bold.

*C* Control Group; *I* Intervention Group, *M* Mean, *SD* Standard deviation, *CI* credible interval, *WCST* Wisconsin Card Sorting Task, *DCCS* Dimensional Change Card Sort test; *GEC* Global Executive Composite, *SDQ* mean teacher and parental rating on the Strengths and Difficulties Questionnaire, *PPP* Posterior Predictive P-value, *LOOIC* Leave-One-Out Information Criterion, *LOO diff* difference in Leave One Out, *SE* standard error.

#### Far transfer|Executive dysfunction

Significant improvements in parent/caregiver-rated executive dysfunction was observed for the intervention group from pre-to-post intervention (*p* < 0.01), and from pre-intervention to follow-up (see Table 4). However, none of the implemented Near to Far transfer sets of models showed evidence of significant change in executive dysfunction from pre-to-post intervention or pre-intervention-to-FU for either group (see Tables 5, 6, 7), nor any difference in the degree of change across groups over time.

#### Far transfer|Social and emotional wellbeing

Significant reductions in parent/caregiver-rated social/emotional problems were observed for the control group from pre-to-post intervention (p < 0.01; see Table 4). However, from pre-intervention to follow-up a significant increase in teacher-rated social/emotional problems were observed for the control group (p < 0.05). None of the implemented sets of Near to Far transfer models showed any evidence of change in social/emotional wellbeing (parent/caregiver/teacher-rated) from pre-to-post intervention or pre-intervention-to-FU for either group (see Tables 5, 6, 7), nor any difference in the degree of change across groups over time.

### Intervention adherence

Students in the intervention group completed on average 12 out of 20 training sessions (range 2 to 20). Seven of the 55 students (13%) were classified as compliers, having completed ≥ 16 training sessions. A sensitivity analysis indicated that compliers and non-compliers did not differ significantly on any of the outcome measures or demographic characteristics at pre-intervention, nor any outcome measures at post-intervention (p > 0.05, data not shown). An additional correlation analysis assessing associations between intervention adherence and intervention effects, revealed no significant relationship between the number of intervention sessions completed and change in the primary outcome (response inhibition) from pre- to post- assessment, r (53) = 0.24, p = 0.083.

### Intervention feasibility

Sixteen educators delivered the intervention within their classrooms. Of these, 12 (75%) completed the feasibility survey (75%) at post-intervention. Educators rated the intervention as feasible (total feasibility score ≥ 3: *M* = 3.42, SD = 0.91) across the following four subscales: acceptability (*M* = 3.50, SD = 0.63), integration (*M* = 3.71, SD = 0.58), adaptability (*M* = 3.25, SD = 0.62) and effectiveness (*M* = 3.00, SD = 0.66). The subscales practicality (*M* = 2.82, SD = 0.85) and implementation (*M* = 2.67, SD = 0.90) were not rated as feasible, with the main barriers to implementation reported as student absence (38.2%) and technical issues (32.7%), with time constraints within the classroom (5.5%), staff shortage (5.5%), and child’s loss of interest (3.6%) also noted.

### Perceived intervention effectiveness

A total of 75 (65%) parent/caregivers completed the perceived effectiveness survey at post-intervention. As shown in Table 8, there were no significant group differences in parental perceived changes in cognition or behaviour between pre- and post-assessment for children allocated to the intervention (*p* > 0.05; total score *M* = 20.65; SD = 3.76) or control groups (*M* = 21.16; SD = 3.66). This lack of significant differences between groups for parental perceived effectiveness was investigated using classification accuracy statistics for parental perceived group allocation. Sensitivity and specificity were both low (0.64 and 0.44 respectively), with around half (55.56%) of the parent/caregiver whose children were allocated to the control incorrectly believing their child was allocated to the intervention. There were no significant differences in parental perceived changes in cognition or behaviour between children whose parent/caregivers believed they were in the intervention (*p* > 0.05; *M* = 21.14; SD = 3.85) compared to children whose parent/caregivers believed they were in the control group (*M* = 20.48; SD = 3.05; see Table 11).

**Table 11:** Means and standard deviations of parent effectiveness ratings per actual treatment group and “perceived” treatment group at post intervention.

	Actual group						Perceived group					
	Control (*n* = 38)		Intervention (*n* = 37)		*Diff*	*p*	Control (*n* = 29)		Intervention (*n* = 43)		*Diff*	*p*
	*M*	*SD*	*M*	*SD*			*M*	*SD*	*M*	*SD*		
Parent effectiveness domain												
Impulsivity	3.42	0.79	3.11	0.84	0.31	0.10	3.14	0.58	3.35	0.92	0.21	0.28
Memory	3.58	0.83	3.57	0.77	0.01	0.95	3.52	0.69	3.65	0.75	0.13	0.45
Cognitive flexibility	3.47	0.80	3.49	0.77	− 0.01	0.94	3.55	0.63	3.49	0.74	− 0.06	0.71
Social skills	3.76	0.82	3.78	0.79	− 0.02	0.91	3.55	0.83	3.88	0.76	0.33	0.08
Emotion regulation	3.39	0.82	3.19	0.88	0.21	0.30	3.24	0.69	3.28	0.93	0.04	0.85
Mental health & wellbeing	3.53	0.89	3.51	0.69	0.01	0.95	3.48	0.63	3.49	0.88	0.01	0.98
Total score	21.16	3.66	20.65	3.76	0.51	0.55	20.48	3.05	21.14	3.85	0.66	0.44

Each domain had a maximum score of 5 with a total maximum score of 30. Higher scores indicate greater perceived improvements from parents.

## Discussion

This pilot randomised controlled trial examined whether a specially designed digital multi-domain cognitive training intervention improved core EFs (inhibitory control, working memory and cognitive flexibility) when compared to teaching-as-usual, among primary school students living in regional areas with high rates of developmental vulnerability and socio-economic disadvantage. Our trial found no evidence that the intervention improved inhibitory control, working memory or cognitive flexibility performance (near transfer) either immediately post-intervention nor at 3-month follow-up. Regarding our secondary aim, there was no evidence that the intervention improved behavioural measures of executive dysfunction or social/emotional wellbeing (far transfer) when compared to teaching-as-usual at any timepoint. Our final aim was to assess the feasibility and acceptability of the program implemented via the school classroom. Despite participating educators rating the digital multi-domain training intervention as feasible and acceptable to implement in the primary school classroom curriculum, compliance with the intervention was low.

This trial is one of few studies to evaluate the impact of school based multi-domain EF training on cognitive and behavioural outcomes in young children. The lack of improvements in trained EFs post-intervention could be considered somewhat surprising given that a recent comprehensive meta-analysis reported that EF training for preschool children (aged 3–6 years old) promoted low-to-medium effects on both near and far EF outcomes^30^. In this meta-analysis of 32 studies, 977 total participants were allocated to intervention groups that administered EF training across at least 10 sessions, with approximately equal numbers of studies investigated EF training via computerised (13 studies, 40.6%) and non-computerised (19 studies, 59.4%) interventions. All but one study included in the meta-analysis involved typically-developing children, with 6 studies (18.8%) including comparisons for children from low socioeconomic backgrounds. Evidence from these studies suggests that EF training effects are greater in children from families with low SES^30^. As such we anticipated positive impacts on EFs in our sample given that children were recruited from areas of the Australian state of Victoria classified as areas of socio-economic disadvantage. However, investigation of the demographic characteristics of our sample indicated that almost half of the students’ parents/caregivers had commenced or completed a university degree or supplementary education beyond secondary school, and the majority were employed. Furthermore, only a very small percentage of students were reported as having a diagnosis of a neurodevelopmental disorder or a learning disability, and the mean IQ of the sample was > 100. The composition of our sample may partially explain the discrepancy between our findings and previous meta-analytical findings^30^. These findings are also are important in highlighting that although samples may be recruited from areas of high developmental vulnerability it does not necessarily ensure that individual participants are developmentally vulnerable.

Another potential reason for the discrepancy in our findings is the older age range of children in the current trial (aged 6–8 years old). Indeed, the authors of the meta-analysis noted that wider transfer effects may occur in pre-schoolers due to EF structures being less defined and as such, training a specific EF more readily impacts other EFs, due to the intercorrelation and overlap of domains at this age^30^. This is supported by developmental research illustrating that key EFs such as response inhibition undergo rapid development in the preschool years, and that subsequent improvements in these skills later in childhood are unlikely to be fundamental changes in cognition but rather refinements in relation to accuracy and efficiency^98,99^. However, this explanation is only relevant to far transfer effects and domains that have not been trained by the intervention, and therefore does not explain the lack of near transfer effects in the current study. Further, positive near transfer effects of training of EFs have been reliably reported in meta-analyses of both primary school children^100^ as well as pre-school children^30^. In fact, meta-analytic findings of primary school children suggest that training multiple rather than single components of EFs in educational practice, is more likely to promote transferable gains to untrained components (i.e. far transfer^100^). As such, we expected even stronger evidence of near transfer in the current study given that multiple EFs were simultaneously targeted through the intervention.

Although several meta-analyses conclude that improvements in near transfer EFs are possible as a result of training, many of the individual trials actually only reported intervention effects on select and not all near outcome measures^30,100,101^. For instance, Kirk et al., (2016) evaluated an attention training program targeting selective and sustained attention in children with neurodevelopmental disorders compared with an active control, and reported near transfer effects on a measure of selective attention, but no effect on a measure of sustained attention^102^. These nuanced findings are often lost when datasets are combined in meta-analytical studies and can lead to distorted reports on the impact of training on targeted outcomes. Indeed when the results of recent individual trials of EF training in neurotypical preschool children^103^ as well as children with developmental vulnerabilities (e.g. neurodevelopmental disorders such as autism spectrum disorder^104^) are reviewed they indicate no near transfer to cognitive measures of EF, consistent with our findings. Furthermore, very few training studies report on children’s progress on the training activities themselves, and fewer still investigate whether change on the training activities are associated with change on near transfer outcome measures^105^. As such when improvements are observed after training it is very difficult to ascertain whether they were the direct result of improved performance on the trained tasks or due to something else entirely.

Another potential explanation for the lack of improvement in near transfer EF tasks examining inhibitory control, working memory and cognitive flexibility is the low compliance with the intervention. Students completed an average of 12 (60%) intervention sessions over the 7-week training period of the trial, with only 13% of students adhering to the required training schedule (i.e. completion of 16 out of 20 intervention sessions). Although a sensitivity analysis indicated there were no differences on any outcome measures between training compliers and non-compliers at either pre or post-intervention, the small number of students who complied (n = 7) meant these comparisons were severely underpowered. Previous studies indicate that the total amount of time spent training is positively and significantly associated with greater improvements in EFs^30,106^. As such it is possible that the limited amount of time spent training in the current trial hampered the effects of the intervention. Future studies that systematically evaluate training dose and training outcomes are needed to elucidate the minimum amount of training time required to promote improvement in EFs. Educators reported that the main barrier to intervention adherence was student absence from the classroom. Low student attendance rates during the time of the trial were not exclusive to our sample, with only 49% of students Australia-wide attending school at least 90% of the time^107^. Despite educational settings offering a supportive and equitable setting to deliver interventions, these findings highlight important limitations (i.e. student absence) that need to be addressed to facilitate the successful implementation of childhood interventions in schools. Future studies that investigate the effect of training schedules (i.e. training conducted at regular intervals vs condensed training) on outcomes measures are needed to assess whether intervention schedules can be adjusted based on student’s attendance without impacting intervention effects.

In relation to far transfer effects (i.e. effect of the training on variables not directly trained) the current trial findings were consistent with our hypotheses that there would be no change in parent/caregiver-reported executive dysfunction or social/emotional wellbeing as a result of multi-domain EF training. These results are consistent with past child studies that highlight efforts to increase EF capacities directly through both single and multi-domain training have generally failed to yield widespread improvements in untrained outcomes such as behaviours, academic achievement and mental health^108^. Although recent meta-analyses have reported improvements on some far transfer measures following multi-domain training^30^, the actual tasks deemed to be assessing far transfer often have significant overlaps with the domains being trained and as such are more likely to be assessing near transfer.

Finally, although educators reported that the intervention was feasible, appropriate for Grade 1 & 2 students, easy to integrate in the classroom and current education system, flexible to diverse needs and had a positive effect on students, some aspects of feasibility (e.g., implementation within the classroom setting, allocated timeframe, and with current resources and training provided) did not meet the required rating threshold. Moreover, students in the intervention group did not meet the required threshold (16 out of 20 sessions on average) to demonstrate adequate intervention compliance. These findings indicate that regardless of educators’ confidence in and perceived capacity to deliver a multi-domain cognitive training program, there is a need for further support to assist educators in motivating students to engage with such programs, even when delivered in a gamified digital format. Previous studies highlighted a number of other relevant barriers (i.e. competing responsibilities, logistics, parental consent/engagement, and administrator/teacher support) that need to be considered when attempting to implement early childhood interventions within the school setting^109^. In cases where these barriers cannot be addressed and student engagement with the intervention is low an alternative setting (i.e. home) may be more viable and efficacious.

The current trial had several strengths, primarily the fact that the multi-domain EF training intervention was specially designed for this trial with involvement from the community including educators, students and Aboriginal people. Further the inclusion of a teaching-as-usual control condition, recruitment of children from developmentally vulnerable regional and remote areas, pre-registration and blinding of researchers were considerable strengths of the trial. However, there are some important limitations that need to be acknowledged. First, compliance with the intervention was low in this pilot trial. Although student absence was reported as the main barrier to compliance, periodic technical issues with the intervention also posed a barrier to engagement. These technical issues were exacerbated by the regional and remote locations of the schools which were > 2 h away from the research hub and therefore difficult to regularly attend in person. Second, we noted a significant amount of missing data for the primary outcome measure (OSARI) which required imputation in order for analysis models to be run. Third, although our sample was appropriately powered to detect a large effect on our outcomes, medium and small effects could have been undetected due to a lack of power. Additionally, the sample was recruited via convenience sampling using predefined characteristics and may therefore lack diversity and not be representative of the greater population. As such the study findings may not be applicable to other primary school locations or populations. Finally, we used adult ratings to measure far transfer skills and understand behavioural change over the course of the trial. We acknowledge these measures are subjective and can suffer from bias and may not be as precise as the objective measures used to assess near transfer. Ideally both subjective and objective measures should be conducted to assess each near and far transfer skill, however this type of data collection needs to be balanced with the time requirements of participants, resources available and the approach taken to data analysis. In conclusion, our findings suggest that digital multi-domain training implemented in the classroom did not have a beneficial effect on EF in primary-school students recruited from regional communities with high rates of developmental vulnerability and socio-economic disadvantage. Despite meta-analytical conclusions^30^, our findings cast some uncertainty around the existence of consistent and significant transfer effects to cognitive or behavioural outcomes following training interventions. Recent commentary in the field has tentatively raised the question of whether enough evidence has been gathered to justify de-implementation of cognitive training interventions in their current form, from clinical and educational practice^108,110^. While our findings seem to concord with these propositions it will be important address the limitations of the present study (e.g., small sample size, sample composition and representativeness, intervention compliance, missing data, use of both objective and subjective outcome measures for each skill), to determine whether digital multi-domain EF training facilitates near (if not far) transfer of EF skills in primary-school children. Although educators deemed the intervention to be feasible, compliance with the intervention was low and educators reported difficulties implementing the intervention for the specified time with their current resources. Given the current poor evidence for the effectiveness of digital multi-domain training, it may be more appropriate to allocate existing educator time, resources and efforts in the classroom to evidence-based tools and practices.

### Supplementary Information

Supplementary Information 1.

Supplementary Information 2.

Supplementary Information 3.

## Data Availability

All data is openly available through the Open Science Framework (https://osf.io/xmjr6/).

## Code availability

Analyses were conducted in R, and all analysis scripts are openly available through the Open Science Framework (https://osf.io/xmjr6/).
