# Supplementary material for: A digital intervention to support childhood cognition after the COVID-19 pandemic: a pilot trial
Source: Sci Rep. 2024 Jun 18;14:14065. doi: 10.1038/s41598-024-63473-2 (PMC11189556; doi:10.1038/s41598-024-63473-2)
Supplement: Supplementary file 1 — Supplementary Information 1. [file 41598_2024_63473_MOESM1_ESM.docx]

# Supplementary information

## Intervention Design | Phase I Design:

School’s eligibility for participation in the study was based on their location in regional Victoria. Due to this criterion, there was a high chance the study would include Aboriginal children as participants. The project team wanted to create the digital training intervention to be inclusive and engaging for any Aboriginal children that participated. To ensure elements of Aboriginal culture were appropriately incorporated into the intervention, an Aboriginal research officer was employed to lead this section of work. An Aboriginal community Elder was consulted to provide feedback and guidance on the naming of the game, design elements to ensure the game was developed with Aboriginal children in mind, and that the design elements fit seamlessly and appropriately into the game. An Aboriginal graphic designer met with the game designer and was contracted to design culturally appropriate elements to fit the game. An Aboriginal musician provided background sounds and music. In addition, two Aboriginal psychology students provided feedback.

## Pilot data | Phase I Design: Educator Interviews and Student Focus Groups

To ensure that the new digital training intervention was suitable and our delivery approach feasible, we conducted focus groups with 77 primary school students (5 to 9 years, mean age = 7.75 years) and collected surveys from 8 Grade 1 and 2 educators from 4 schools across Victoria (June – December 2020). Educators completed an initial survey that asked about challenges in the classroom, digital technology use, and cognitive training programs. Educators indicated that the biggest challenges that students in their classroom faced were poor concentration (62.5%), unique needs of students (e.g., neurodevelopmental disorders or trauma; 37.5%), lack of availability of technology (25%), ranging abilities of students (25%) and lack of parent support (25%). Of the educators surveyed, 87.5% said problems in cognitive skills had a major or large impact on learning. Difficulties with memory and inhibition were rated as the most problematic cognitive weaknesses. Despite these difficulties, when asked if they had the resources to support cognitive development in childhood, 50% of educators responded, “*not at all*” and 50% responded “*somewhat.*” Further, although 87.5% of educators thought it was important for them to be involved in the development of school-based cognitive interventions, none of the surveyed educators had previously been approached with this opportunity. All educators surveyed had access to technology and the internet, had previously used technology to support their teaching, and found these digital tools useful. 50% of educators used digital technology more than once a day, 25% once a day, 12.5% 1-2 times a week and 12.5% less than once a week. Educators rated curriculum demands as the single most important factor which would determine their use of digital training interventions.

## Pilot data | Phase IIa: Proof-of-Concept Trial

To assess the feasibility and viability of the school-based intervention in improving executive functioning, as well as the trial protocol, a proof-of-concept study was conducted. A total of 18 Grade 1 students (aged 6 years to 7 years 1 month; 12 males) were recruited from 3 classes from a primary school in the state of Victoria, Australia. All participating students completed cognitive assessments of inhibitory control, working memory and cognitive flexibility prior to commencing the digital intervention. The assessment measures are the same as the primary and secondary outcome measures included in the current protocol for the Phase IIb: Pilot Trial. The intervention was completed for 30 minutes during class-time 2-3 times a week, over a 7-week period during Term 2, 2022 (May-June). All the cognitive assessments were conducted again immediately after the 7-week intervention period. One participant withdrew from the study at post-training due to parent concerns about time spent away from other educational activities. Results indicated that all students were able to complete each of the outcome measures with no floor or ceiling effects, and that students were able to engage with the intervention independently in the classroom. Over the 7-week intervention period students completed an average of 5 out of a possible 20 training sessions (27%), indicating significant concerns with intervention compliance. Therefore, for the *Phase IIb: Pilot Trial* specific dates and times will be agreed with educators to complete the intervention and researchers will be present for 50% of the training sessions to support educators in the implementation of the intervention during class time.

## Educator Feasibility Survey | Phase IIb: Pilot Trial

Please read the following statements and answer by selecting the option that best describes your opinion.

|  |  | Completely disagree | Disagree | Neither agree nor disagree | Agree | Completely agree |
| --- | --- | --- | --- | --- | --- | --- |
| **Acceptability** | | | | | | |
|  | Caterpillar Creek meets my approval | ➀ | ➁ | ➂ | ➃ | ➄ |
|  | Caterpillar Creek is appealing to me | ➀ | ➁ | ➂ | ➃ | ➄ |
|  | I would let my students use Caterpillar Creek again | ➀ | ➁ | ➂ | ➃ | ➄ |
|  | Caterpillar Creek was appropriate for Grade 1 and Grade 2 students | ➀ | ➁ | ➂ | ➃ | ➄ |
|  | Students found Caterpillar Creek engaging | ➀ | ➁ | ➂ | ➃ | ➄ |
|  | Caterpillar Creek had a positive impact on our Class | ➀ | ➁ | ➂ | ➃ | ➄ |
|  | Caterpillar Creek did not cause any adverse side effects (e.g., headaches, eye strain, dizziness) | ➀ | ➁ | ➂ | ➃ | ➄ |
| **Practicality** | | | | | | |
|  | Caterpillar Creek was easy to implement | ➀ | ➁ | ➂ | ➃ | ➄ |
|  | The duration of each Caterpillar Creek session (20 mins) was suitable | ➀ | ➁ | ➂ | ➃ | ➄ |
|  | The duration of the training period (7 weeks) was suitable | ➀ | ➁ | ➂ | ➃ | ➄ |
|  | Students were able to successfully complete Caterpillar Creek in the classroom | ➀ | ➁ | ➂ | ➃ | ➄ |
|  | Caterpillar Creek could be implemented with the schools’ current resources | ➀ | ➁ | ➂ | ➃ | ➄ |
|  | Caterpillar Creek could be implemented with the training/information provided | ➀ | ➁ | ➂ | ➃ | ➄ |
| **Integration** | | | | | | |
|  | Caterpillar Creek could be integrated into classroom activities | ➀ | ➁ | ➂ | ➃ | ➄ |
|  | Caterpillar Creek is aligned with the current education system | ➀ | ➁ | ➂ | ➃ | ➄ |
| **Adaptability** | | | | | | |
|  | Caterpillar Creek is flexible and accommodates diverse needs. | ➀ | ➁ | ➂ | ➃ | ➄ |
| **Implementation** | | | | | | |
|  | Caterpillar Creek was successfully implemented in the classroom. | ➀ | ➁ | ➂ | ➃ | ➄ |
|  | I was able to run Caterpillar Creek as intended (20 sessions over 7 weeks). | ➀ | ➁ | ➂ | ➃ | ➄ |
|  | Caterpillar Creek was easy to use. | ➀ | ➁ | ➂ | ➃ | ➄ |
|  | The teacher’s manual contained all the information I needed to implement Caterpillar Creek. | ➀ | ➁ | ➂ | ➃ | ➄ |
|  | The commitment required by me was realistic. | ➀ | ➁ | ➂ | ➃ | ➄ |
| **Effectiveness** | | | | | | |
|  | Caterpillar Creek had a positive effect on students | ➀ | ➁ | ➂ | ➃ | ➄ |
|  | Caterpillar Creek improved students’ inhibitory control (e.g., impulsivity) | ➀ | ➁ | ➂ | ➃ | ➄ |
|  | Caterpillar Creek improved students’ working memory (e.g., remembering) | ➀ | ➁ | ➂ | ➃ | ➄ |
|  | Caterpillar Creek improved students’ cognitive flexibility (e.g., switching between tasks) | ➀ | ➁ | ➂ | ➃ | ➄ |

## Phase IIb: Pilot Trial | Detailed Description of Analytical Analysis

Latent change score models [89] were used to estimate within and between group variance at once as well as sequential effects of variables on each other over time and an unobserved latent change score. As per McArdle and Prindle [90], separate analyses were conducted to compare pre-intervention to post-intervention outcomes, and then pre-intervention to follow-up (FU) outcomes. Separate comparisons were conducted for each executive functioning domain (inhibitory control, working memory and cognitive flexibility) resulting in three sets of model comparisons (see Tables 5 to 7). As Bayesian model comparison was utilised credible intervals (99%) are used rather than p-values [100]. To set up each model, a Near latent common factor (N0) was created comprising scores on the relevant Near transfer outcome measures at pre-intervention, and a post-test Near latent common factor was created (N1), comprising scores on the same outcome measures at post-intervention. Similarly, two Far latent factors were created, comprising scores on the Far transfer measures (Executive Dysfunction and Social/emotional Wellbeing), measured at pre-test (F0) and post-test (F1). The unobserved changed component was modelled by including a Near Change (NC), and a Far Change (FC) simultaneous regression, each predicted by N0 and F0, thereby allowing autoregression and lagged and cross-lagged regression to be incorporated into the model, so that the sequential effect of each variable upon other variables and timepoints was included in the model. The post-test common factor scores (N1 and F1) were modelled as pre-test scores plus the unobserved change component with fixed-unit values (N1 = 1*N0 + 1*NC, and F1 = 1*F0 + 1*FC). In all analyses an average Social/emotional Wellbeing score which combined the mean of both parent’s and the teacher’s ratings was used to avoid issues of multicollinearity.

As per Figure 4 in McArdle and Prindle [90], ‘group’ (i.e. intervention vs control group) was not included as a model factor. Rather, the models were implemented upon both groups at the same time by using the ‘group’ property in blavaan. The pre-post comparison was implemented four times (and, similarly, the pre-follow-up comparison was implemented four times, so eight models were implemented for each executive function domain). The degree of measurement invariance was increasingly relaxed across the groups for each model, and then model fit was compared. The level of invariance across the groups of the model that provides the best fit indicates group differences, as explained further below. All models required fixed factor loadings across the groups, so that the weighting of each observed variable upon each factor is equal across both groups. The increasing relaxation of invariance across the four models is now described, as well as what each model would show, should it be the best fitting of the four models.

Model 1 is the simplest and most invariant model, requiring fixed regression coefficients and intercepts to be invariant over time and groups. If this model fits better than the other models, the model indicates no differences between groups on any parameters, and no change over time.

Model 2 relaxed Model 1 further by allowing the change intercepts to differ over groups (i.e. the intercepts of NC and FC). If this model is superior to Model 1, it suggests that there may be differences in the Near Change and Far Change latent factors between groups, but no evidence that the degree of change is different across groups. This is similar to observing a difference in pre and post scores for each group in a traditional RCT ANOVA, but not an interaction between groups over time.

Model 3 allowed the pre-test transfer regression (i.e. N0 + F0 🡪 NC and N0 + F0 🡪 FC) to differ over groups, that is, the impact of the autoregression, and lagged and crossed regressions. If this model is superior to Model 2, this suggests that the dynamic and sequential effects of variables within the model differ between groups.

Finally, for Model 4 the Near Change to Far Change regression is allowed to differ across groups. If this model fits the best, this suggests that the transfer of change from Near to Far differs between groups, equivalent to a group by time interaction in a traditional 2x2 mixed-ANOVA in an RCT design.

All models were implemented using the blavaan package for Bayesian latent variable analysis in R [101] that relies on JAGS and Stan to estimate models via MCMC. Prior to implementing models, all variables were standardised to prevent excessively large covariates impacting model convergence. There were no issues with floor or ceiling effects for any of the outcome measures and intra-cluster coefficients were very small between classroom and other variables (ranging from 0.002 to 0.24; intra-cluster coefficients compared variance between and within clusters for each Near and Far transfer variable for teacher groups, ignoring treatment groups and timepoint).

Posterior Predictive P-values (PPP) were used to assess model fit, with values close to 0.5 indicating good fit [94]. In addition, all models had issues with neither diagonal nor unrestricted theta covariance matrices., which estimates the portion of the variance in the observed variable not explained by the latent variables. This issue is very complicated to manage in Bayesian models. Given this, Leave-One-Out Information Criterion (LOOIC) was used to compare model fit, rather than Bayes factors, as the former does not rely on estimates of the priors and so is less affected by issues with the theta covariance matrix than the latter. The lower the LOOIC value, the better the model fit. A LOO discrepancy of 2 to 4 can be interpreted as a moderate difference in model fit, while a LOO discrepancy of greater than 4 can be interpreted as a large difference.

## Model implementation

Default priors set by the Stan package in R were used (which are used by blavaan), except for the intercepts of the observed and latent variables, and the precision of the observed variables. The intercepts were set to normal(0,1), as the data were standardised to assist with model convergence. A gamma distribution was set on the precision, with df=0.20,0.5.

When running the models, three Markov-chains were converged to estimate the posterior probabilities, each with 6,000 iterations, 1000 burnins, and thinning of 10 to speed up processing. Convergence was examined with trace plots, Gelman-Rubin and Geweke diagnostics (the upper limit of the within and between chain variability is around 1, and z-scores for a test of equality of means between the first and last parts of each chain were within +/-1.96), r-hat less than 1.05, effective sample size of at least 700 (i.e. 10% of the iterations), and MCSE less than 10% of the posterior standard deviation. All diagnostic criteria were satisfied, indicating convergence of all models. However, as noted above, all models had issues with a non-diagonal or non-restricted theta covariance matrix (which represents the error variances and covariances in the model) which may impact the estimate of the priors.

To understand whether the current findings were the product of modelling decisions we also examined latent growth curves as well as hierarchical linear models (Bayesian and Conventional), however the reported results remained the same.
